# Supplementary material for: Oncogenic YAP sensitizes cells to CHK1 inhibition via CDK4/6 driven G1 acceleration
Source: EMBO Rep. 2025 Jul 4;26(16):4017–39. doi: 10.1038/s44319-025-00514-5 (PMC12373906; doi:10.1038/s44319-025-00514-5)
Supplement: Supplementary file 1 — Table EV1 [file 44319_2025_514_MOESM1_ESM.pdf]

**Table EV1: Oligonucleotide and siRNA sequences**

**RT-qPCR primers**

| Gene | fw primer | bw primer |
|---|---|---|
| AMOTL2 | AGGCTGCAGAGAGACAATGAG | CTCAGAGAGCCGCTGGATT |
| CCNA2 | GGTACTGAAGTCCGGGAACC | GAAGATCCTTAAGGGGTGCAA |
| CCND1 | GGCGGAGGAGAACAAACAGA | GGAGGGCGGATTGGAAATGA |
| CCNE1 | AGAGGAAGGCAAACGTGACC | GGGTCTGCACAGACTGCAT |
| CDC20 | CTGTCTGAGTGCCGTGGAT | TCCTTGTAATGGGGAGACCA |
| CDK4 | ACACCCGTGGTTGTTACACT | TCGGCTTCAGAGTTTCCACA |
| CDK6 | AAGTCTTGCTCCAGTCCAGC | CTGGGAGTCCAATCACGTCC |
| FANCD2 | AAGTCGAAAACTACGGGCGG | GGCATCTTCTGTCAGGCTCT |
| KIF23 | CCTAACGTCCCGCAGTCTT | AGGTTTCCGGGGTGTCTTAG |
| MYBL2 | TCCACACTGCCCAAGTCTCT | AGCAAGCTGTTGTCTTCTTTGA |
| PKMYT1 | CACCACTGAGGCTGTTGGG | GAAGTAGGCTGGGACTGGGA |
| RRM2 | TGCGTCGATATTCTGGCTCA | TCCTCCGATGGTTTGTGTACC |
| TK1 | GTCATAGGCATCGACGAGGG | CCAGTGCAGCCACAATTACG |
| TMYS | CTGGGGCAGATCCAACACAT | CACACGTTTGGTTGTCAGCA |
| WEE1 | AAGCTGCGACTCTTCGACAC | AGCTGGAATCAATTCCCCGA |

**siRNAs**

| Gene | Name/ sequence | Reference |
|---|---|---|
| ctrl | Cat#4390843 | Thermo Fisher Scientific |
| WEE1 | CAUCUCGACUUAUUGGAAAtt | |
| PKYMT1 | GGACAGCAGCGGAUGUGUUtt | |
