# Supplementary figures and images for: Oncogenic YAP sensitizes cells to CHK1 inhibition via CDK4/6 driven G1 acceleration
Source: EMBO Rep. 2025 Jul 4;26(16):4017–39. doi: 10.1038/s44319-025-00514-5 (PMC12373906; doi:10.1038/s44319-025-00514-5)

Figure 2I

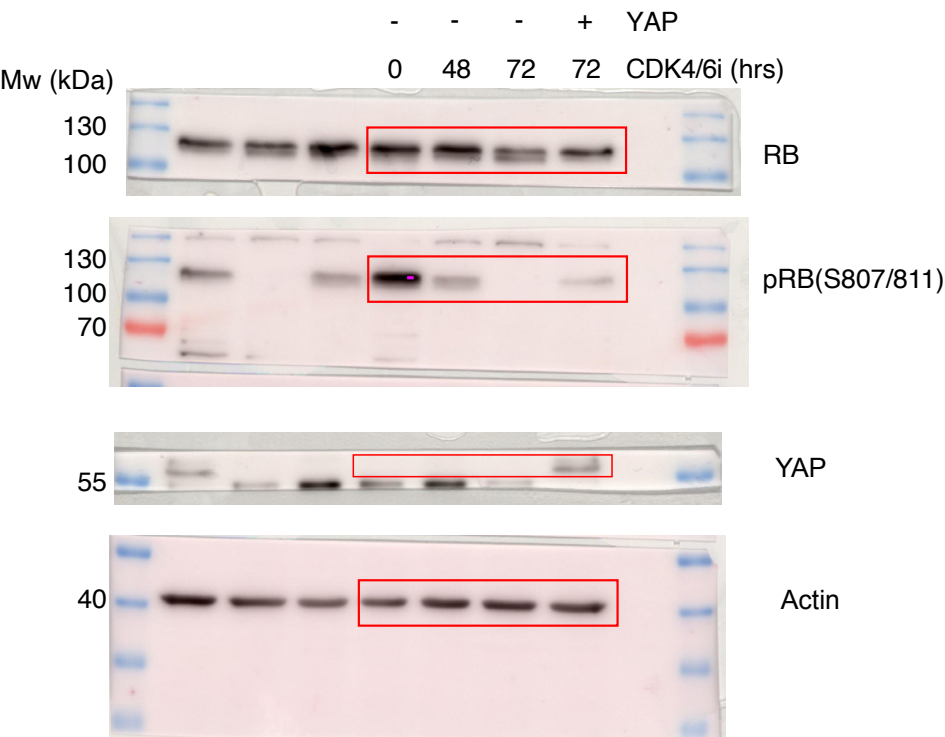

Supplement: Supplementary file 4 — Source data Fig. 2 [file 44319_2025_514_MOESM4_ESM.zip › Figure 2/2I/western blots_Figure2I.pdf]

Figure 2F

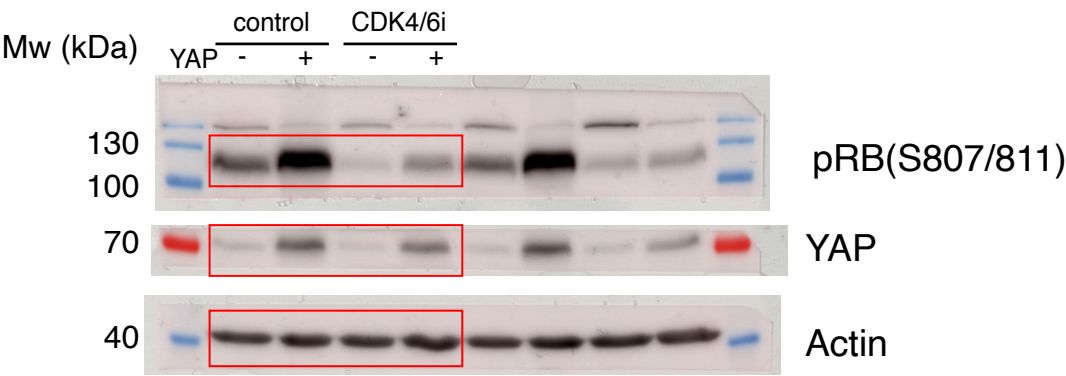

Supplement: Supplementary file 4 — Source data Fig. 2 [file 44319_2025_514_MOESM4_ESM.zip › Figure 2/2F/Western_blots_2F.pdf]

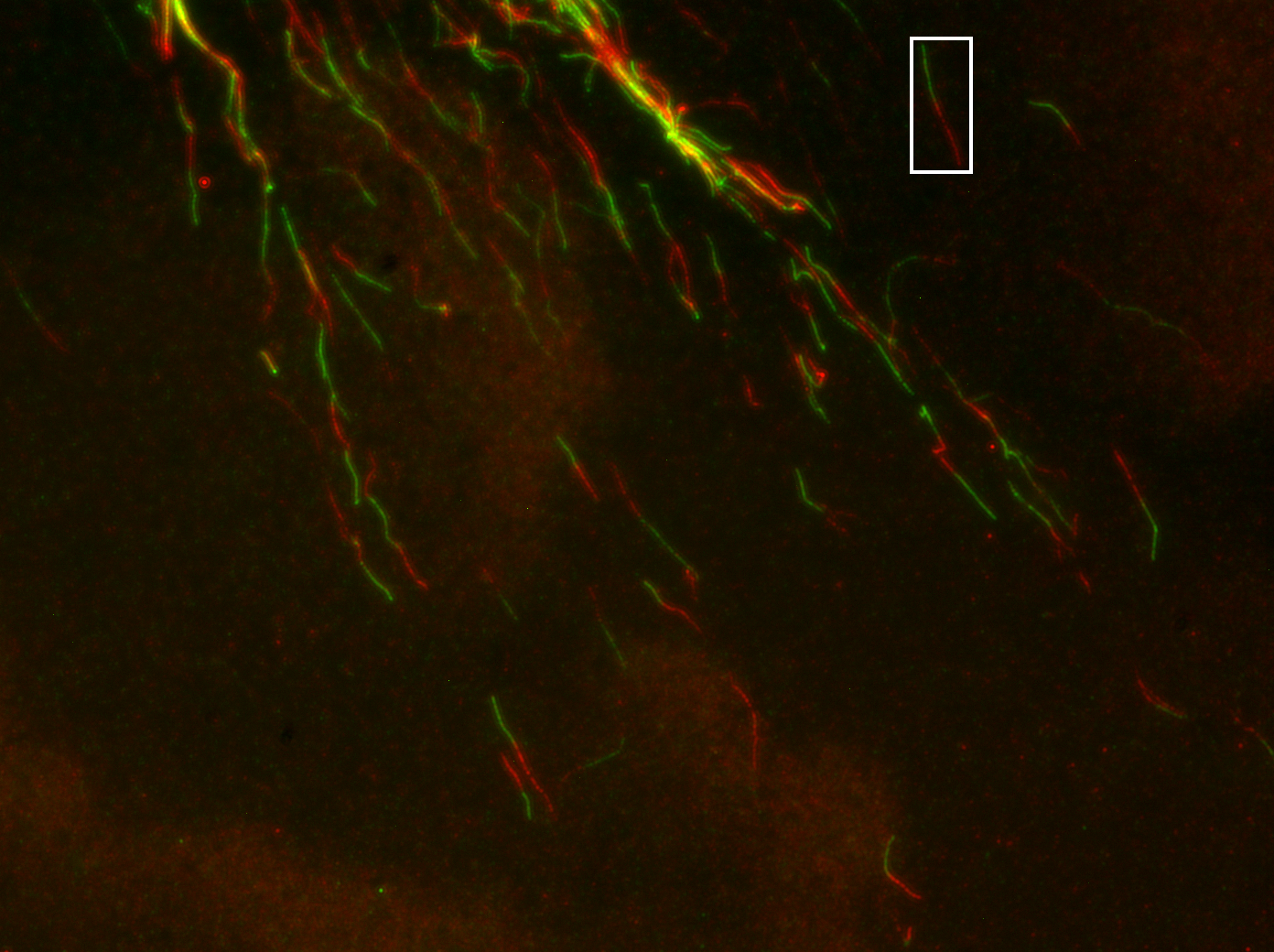

Supplement: Supplementary file 5 — Source data Fig. 3 [file 44319_2025_514_MOESM5_ESM.zip › Figure 3/3F/fibre_assay_YAP.tif]

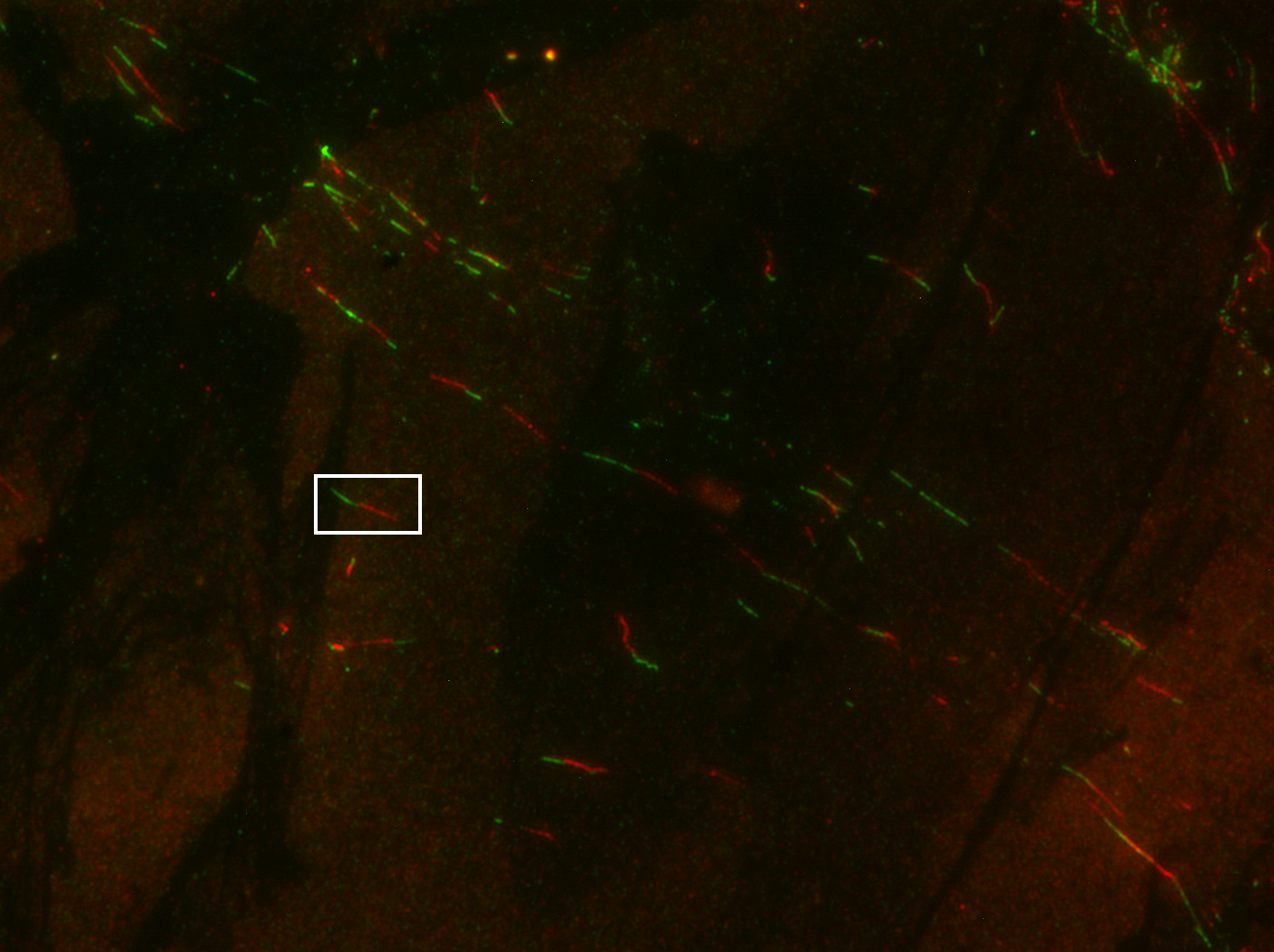

Supplement: Supplementary file 5 — Source data Fig. 3 [file 44319_2025_514_MOESM5_ESM.zip › Figure 3/3F/fibre_assay_YAP+CHK1i.tif]

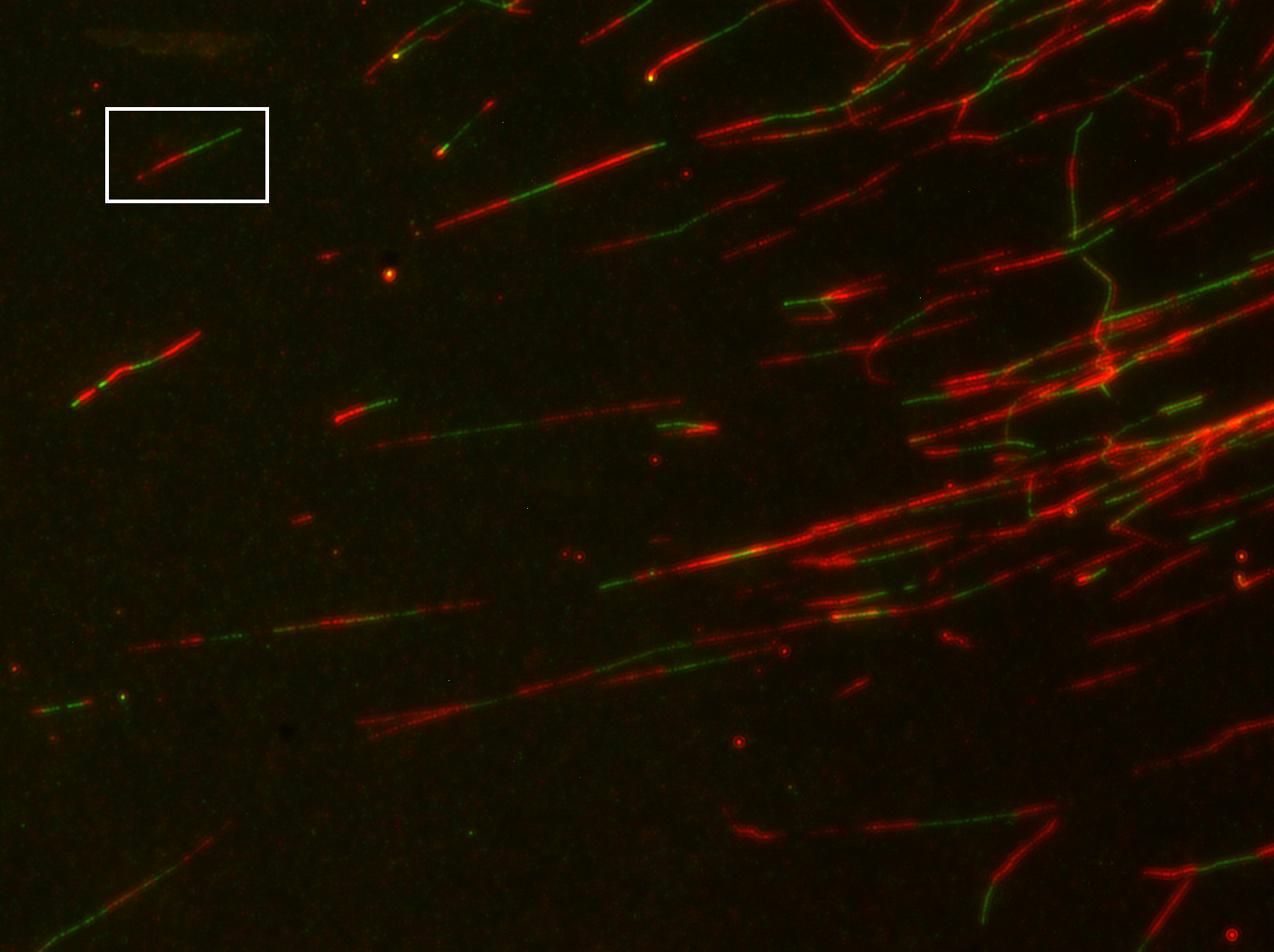

Supplement: Supplementary file 5 — Source data Fig. 3 [file 44319_2025_514_MOESM5_ESM.zip › Figure 3/3F/fibre_assay_ctrl.tif]

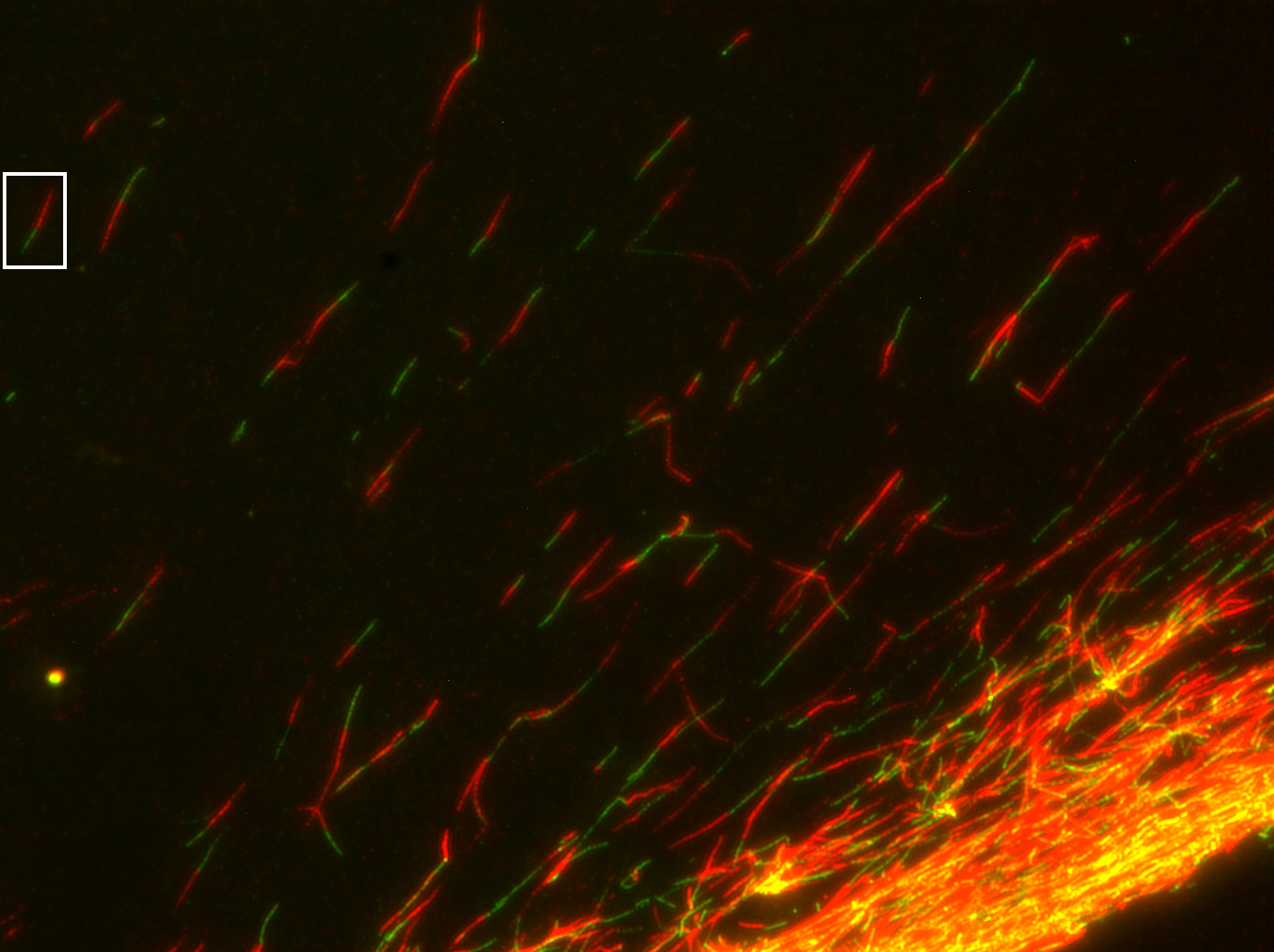

Supplement: Supplementary file 5 — Source data Fig. 3 [file 44319_2025_514_MOESM5_ESM.zip › Figure 3/3F/fibre_assay_CHK1i.tif]

Figure 4A

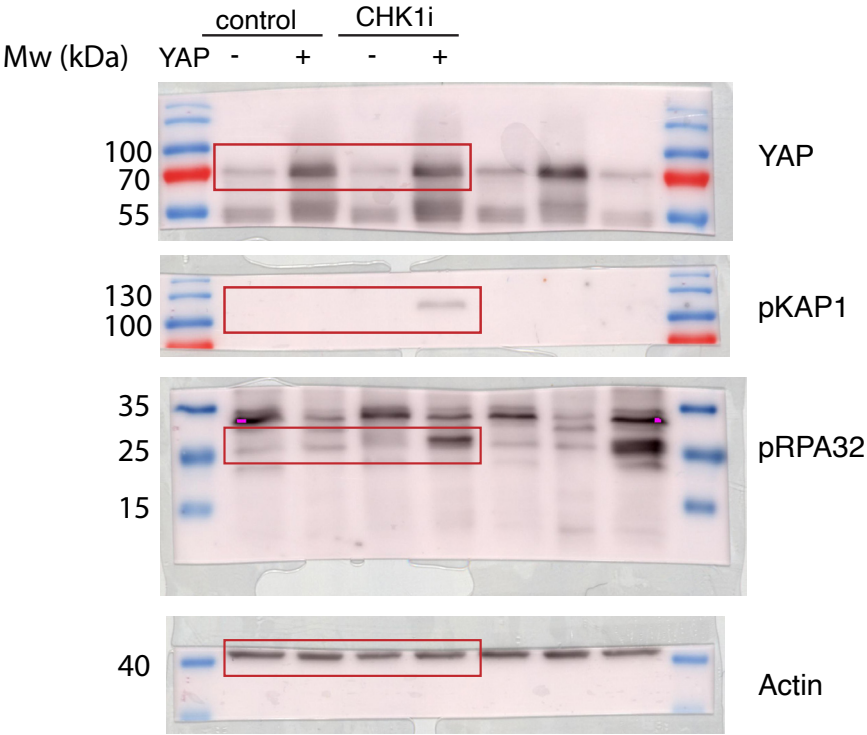

Supplement: Supplementary file 6 — Source data Fig. 4 [file 44319_2025_514_MOESM6_ESM.zip › Figure 4/4A/western_4A.pdf]

Figure 5F

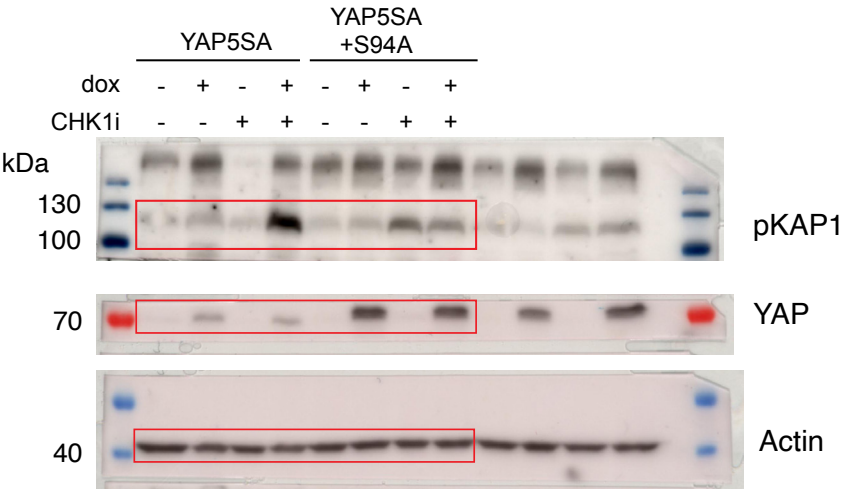

Supplement: Supplementary file 7 — Source data Fig. 5 [file 44319_2025_514_MOESM7_ESM.zip › Figure 5/5F/westerns_5F.pdf]

Figure 5J

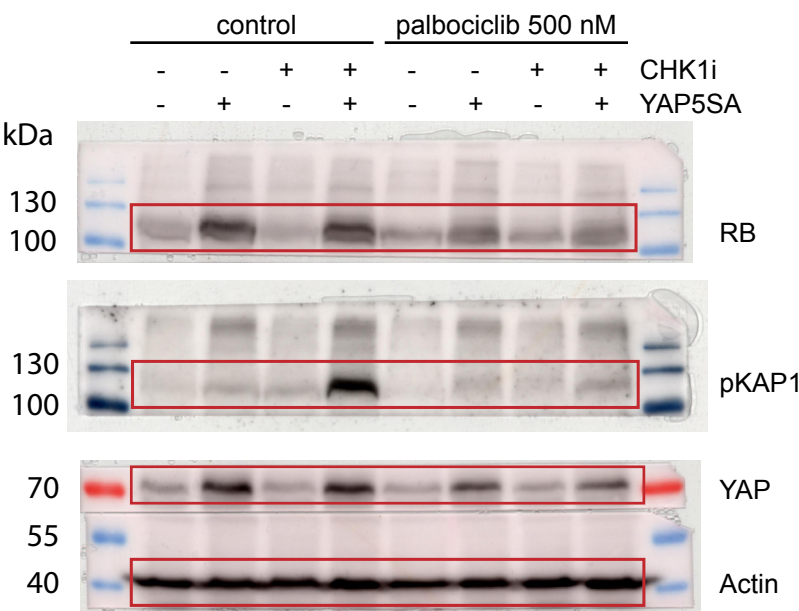

Supplement: Supplementary file 7 — Source data Fig. 5 [file 44319_2025_514_MOESM7_ESM.zip › Figure 5/5J/westerns_5J.pdf]

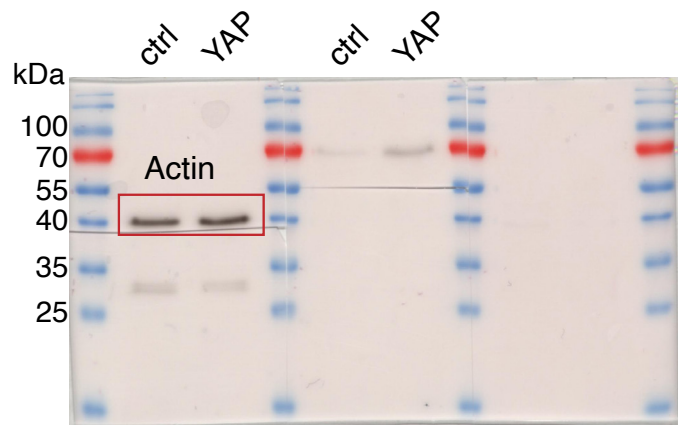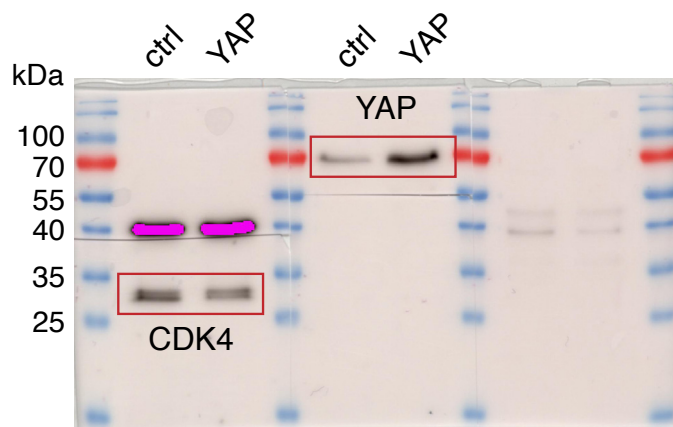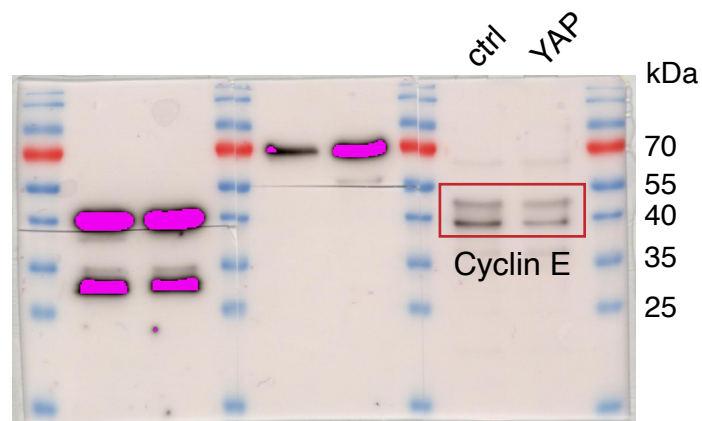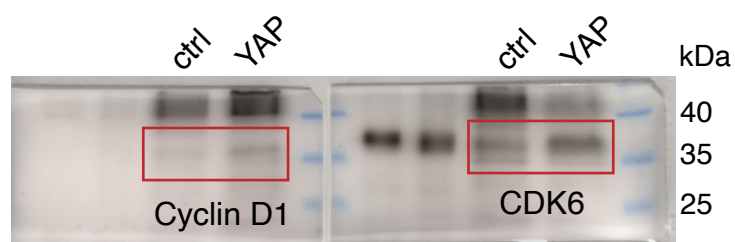

Supplement: Supplementary file 8 — Figure EV1 Source Data [file 44319_2025_514_MOESM8_ESM.zip › EV1/EV1G/westerns_EV1G.pdf]

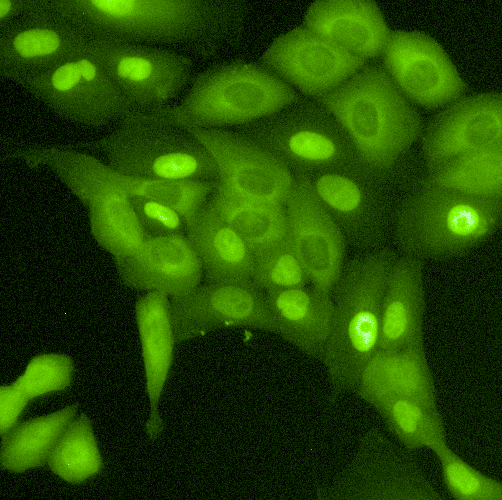

Supplement: Supplementary file 8 — Figure EV1 Source Data [file 44319_2025_514_MOESM8_ESM.zip › EV1/EV1A/CDK2.tif]

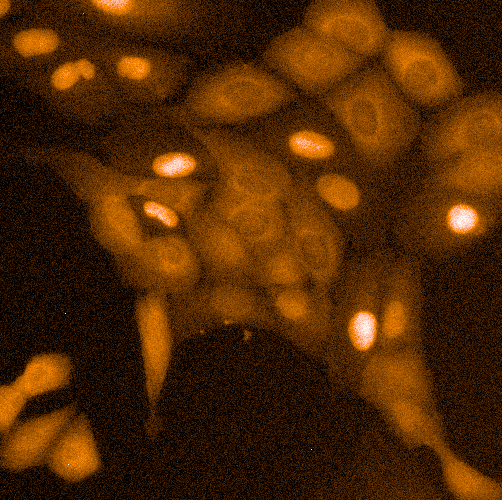

Supplement: Supplementary file 8 — Figure EV1 Source Data [file 44319_2025_514_MOESM8_ESM.zip › EV1/EV1A/CDK4.tif]

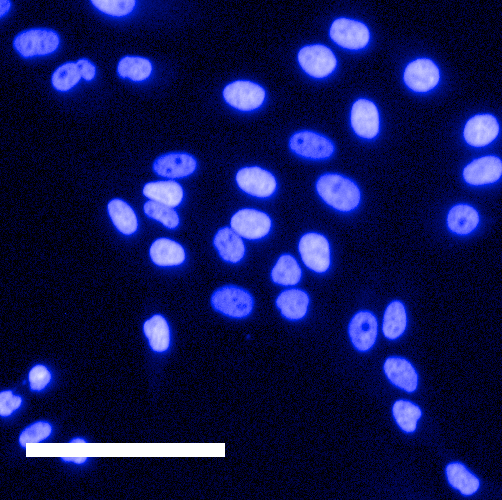

Supplement: Supplementary file 8 — Figure EV1 Source Data [file 44319_2025_514_MOESM8_ESM.zip › EV1/EV1A/Hoechst.tif]

Figure EV2B

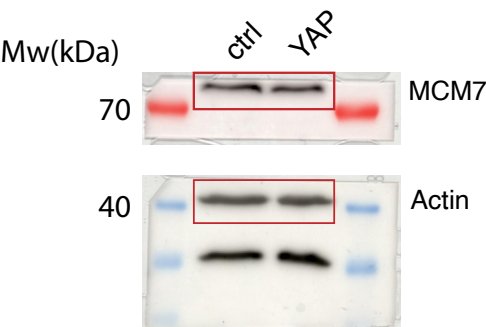

Supplement: Supplementary file 9 — Figure EV2 Source Data [file 44319_2025_514_MOESM9_ESM.zip › EV2/EV2B/westerns_EV2B.pdf]

Figure EV2D

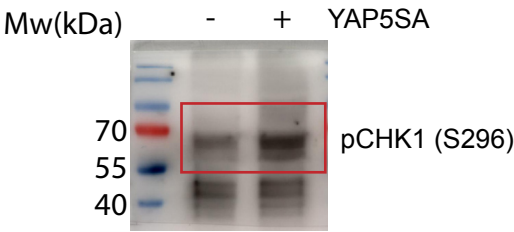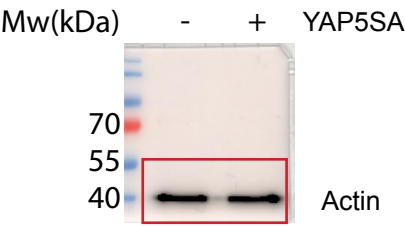

Supplement: Supplementary file 9 — Figure EV2 Source Data [file 44319_2025_514_MOESM9_ESM.zip › EV2/EV2D/westerns_EV2D.pdf]

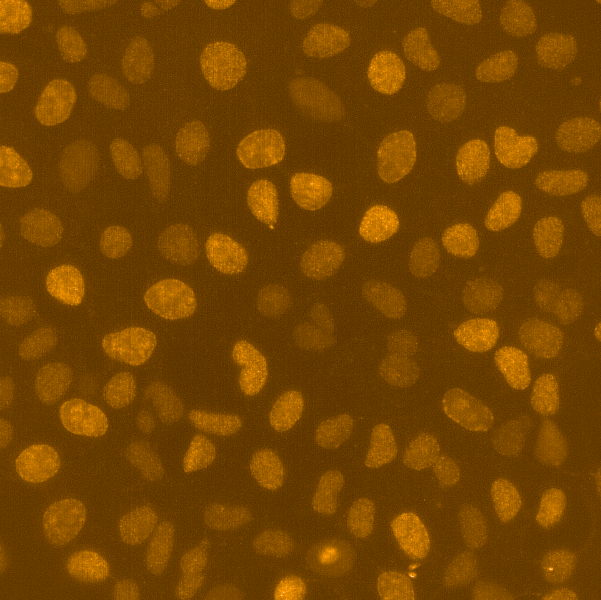

Supplement: Supplementary file 9 — Figure EV2 Source Data [file 44319_2025_514_MOESM9_ESM.zip › EV2/EV2A/MCM7.tif]

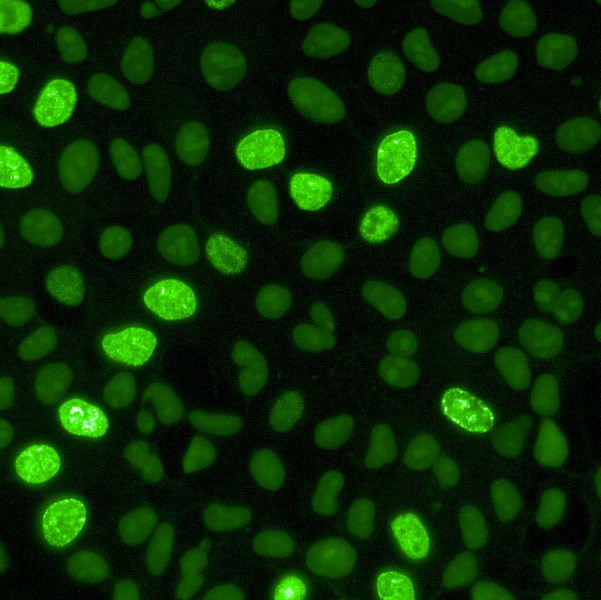

Supplement: Supplementary file 9 — Figure EV2 Source Data [file 44319_2025_514_MOESM9_ESM.zip › EV2/EV2A/EdU.tif]

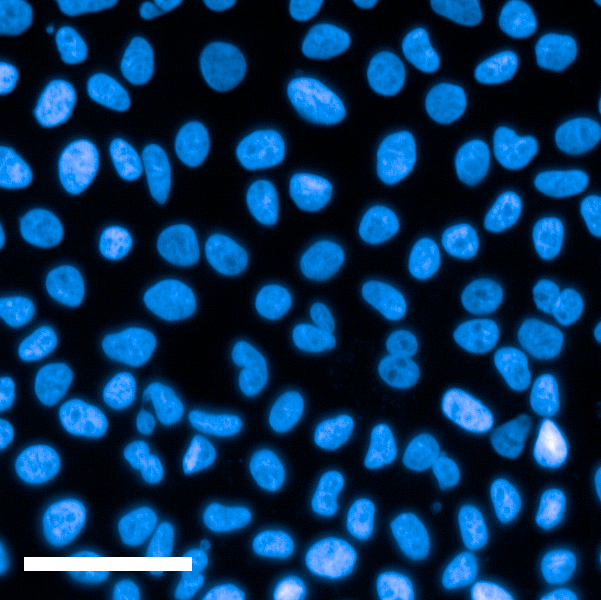

Supplement: Supplementary file 9 — Figure EV2 Source Data [file 44319_2025_514_MOESM9_ESM.zip › EV2/EV2A/Hoechst.tif]

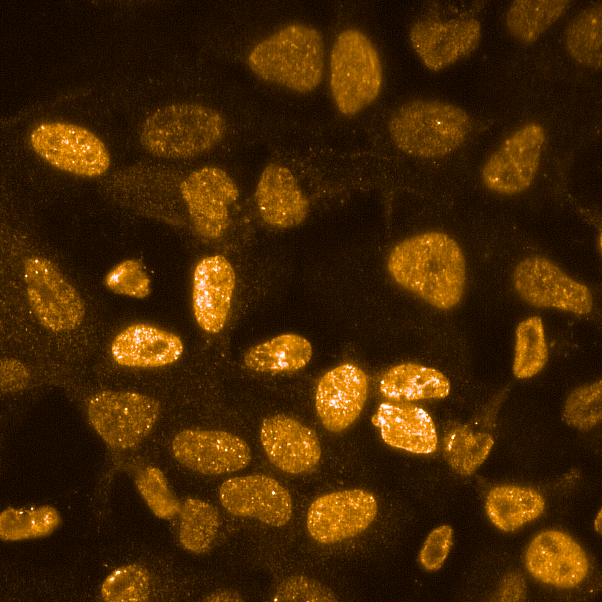

Supplement: Supplementary file 10 — Figure EV3 Source Data [file 44319_2025_514_MOESM10_ESM.zip › EV3/EV3A/gammaH2AX.tif]

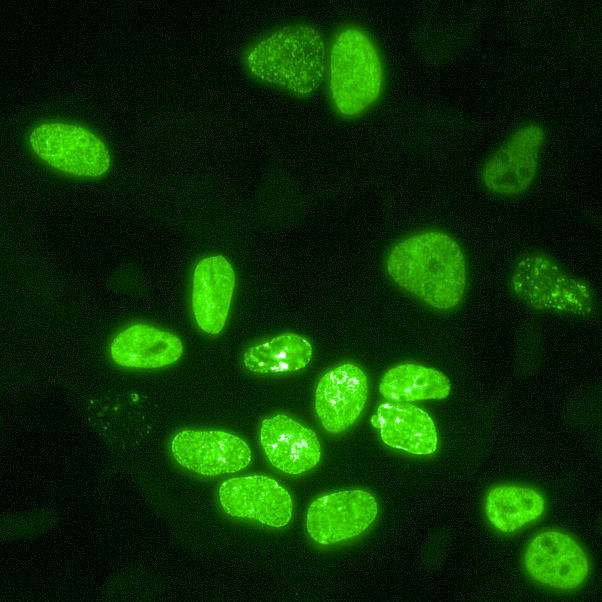

Supplement: Supplementary file 10 — Figure EV3 Source Data [file 44319_2025_514_MOESM10_ESM.zip › EV3/EV3A/EdU.tif]

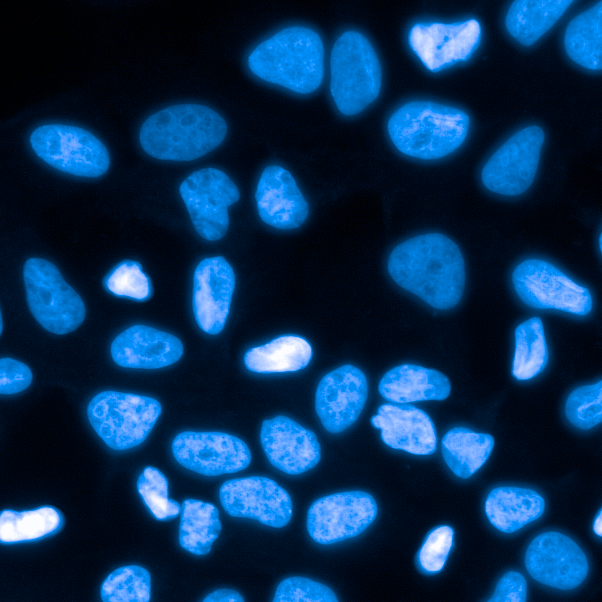

Supplement: Supplementary file 10 — Figure EV3 Source Data [file 44319_2025_514_MOESM10_ESM.zip › EV3/EV3A/Hoechst.tif]

Figure EV4D

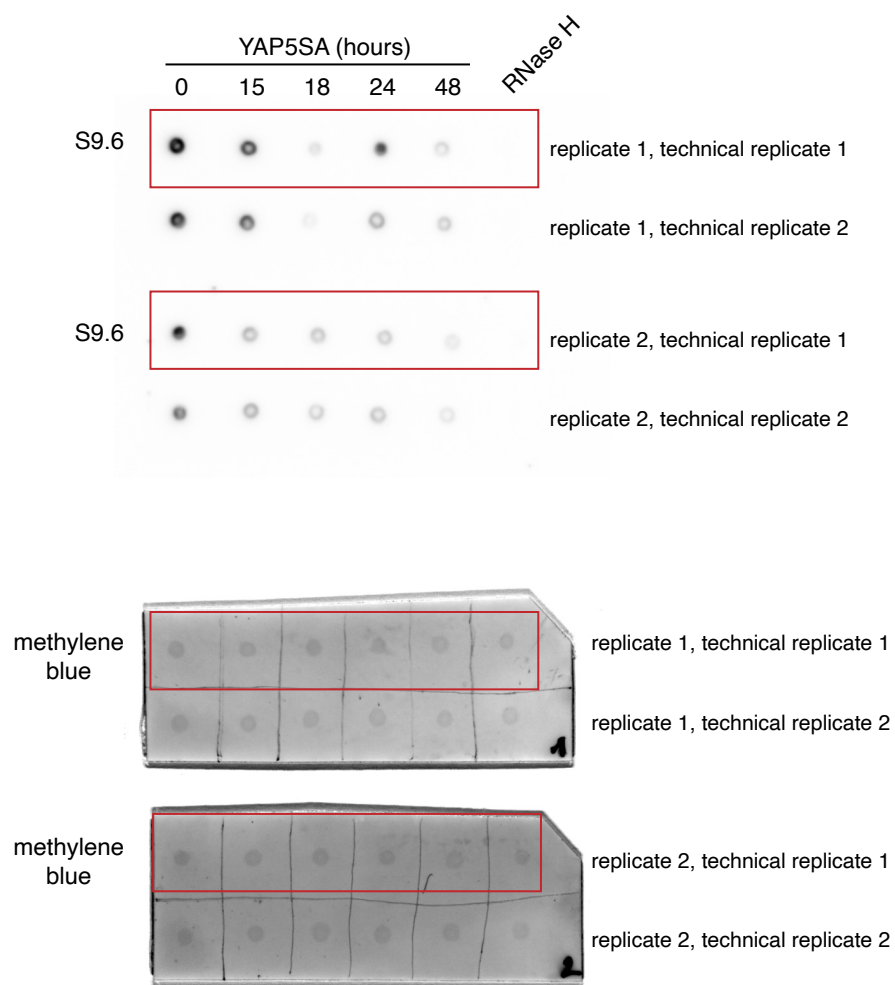

Supplement: Supplementary file 11 — Figure EV4 Source Data [file 44319_2025_514_MOESM11_ESM.zip › EV4/EV4D/S9.6_dot_blots.pdf]

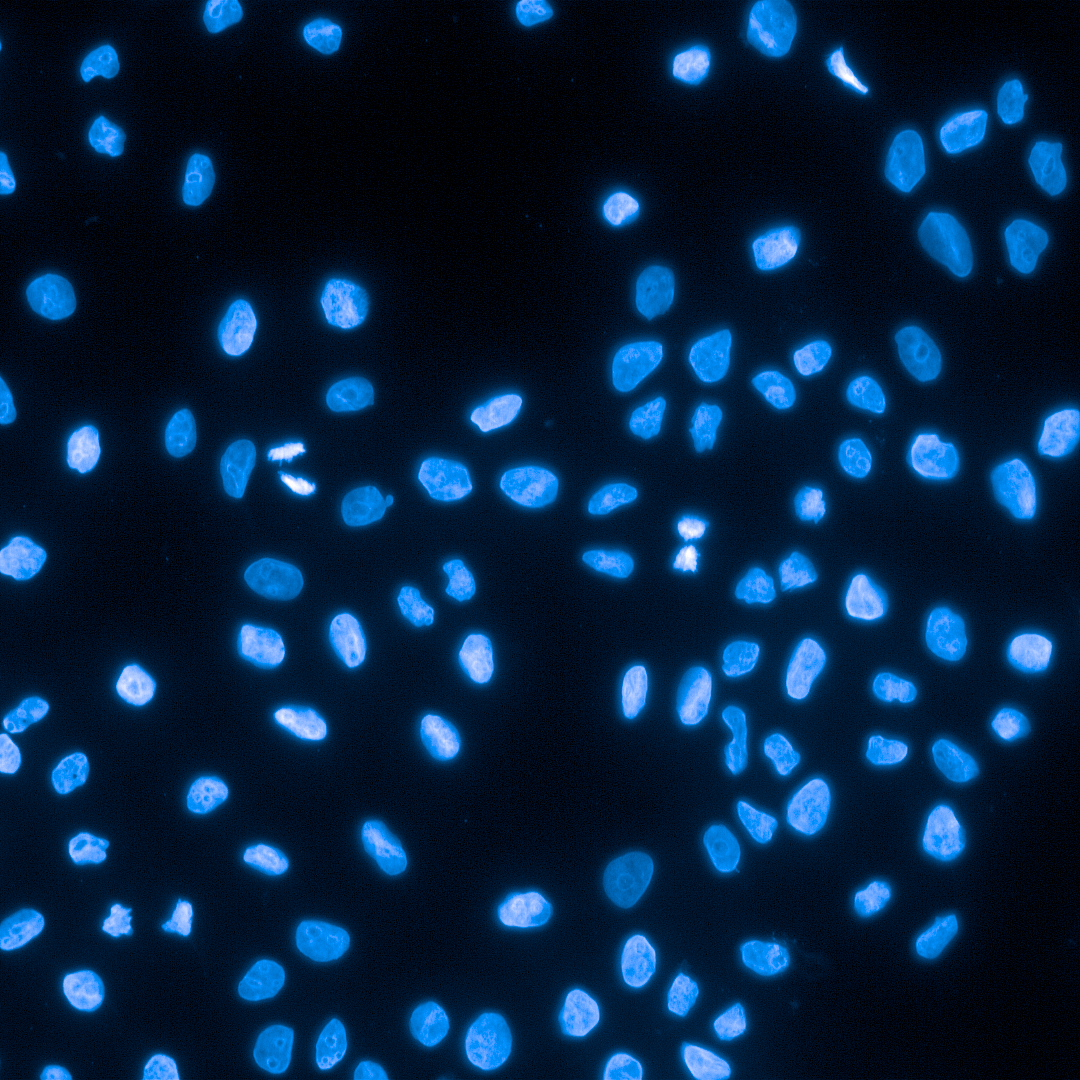

Supplement: Supplementary file 11 — Figure EV4 Source Data [file 44319_2025_514_MOESM11_ESM.zip › EV4/EV4C/CHK1i_40_Hoechst.tif]

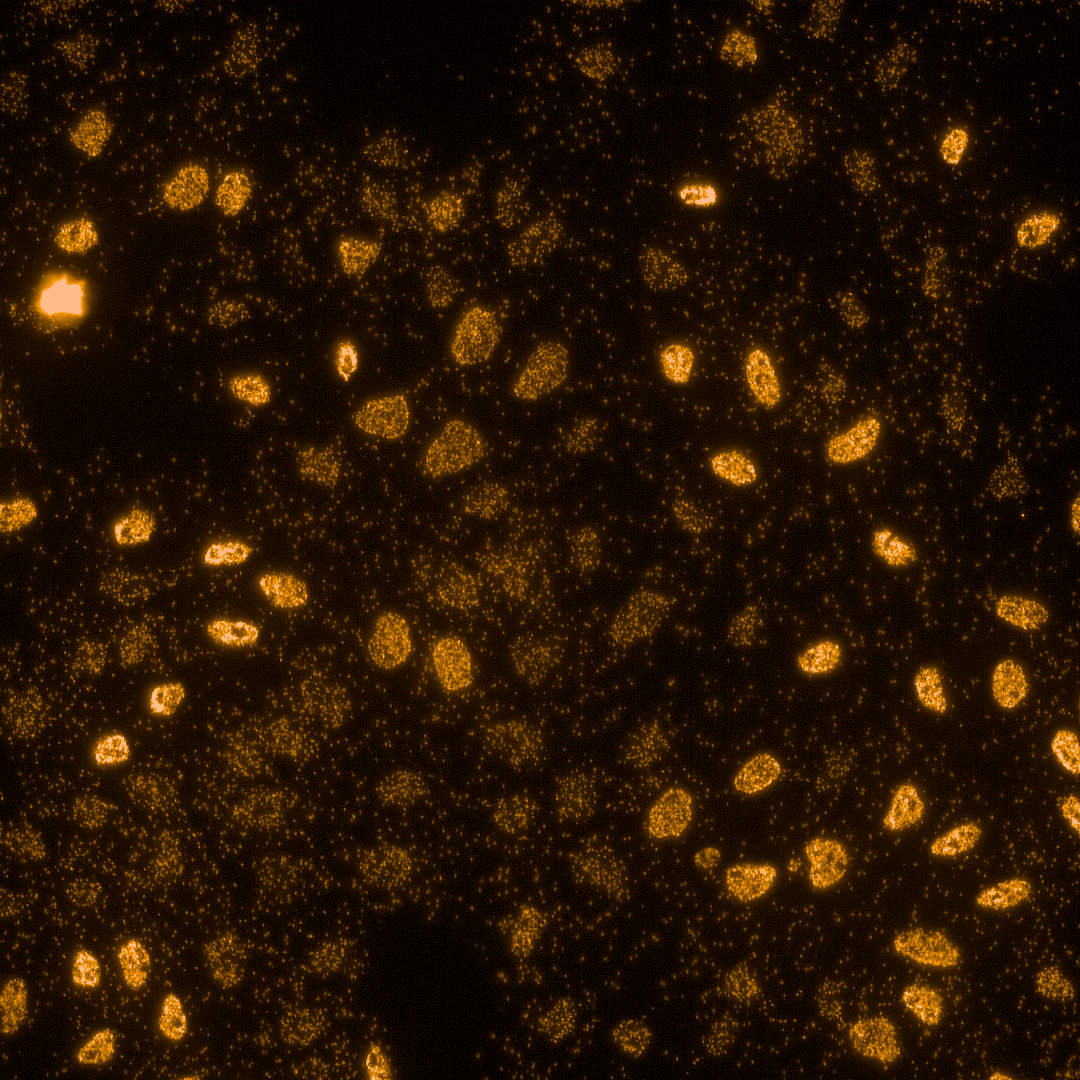

Supplement: Supplementary file 11 — Figure EV4 Source Data [file 44319_2025_514_MOESM11_ESM.zip › EV4/EV4C/DMSO_45_PLA.tif]

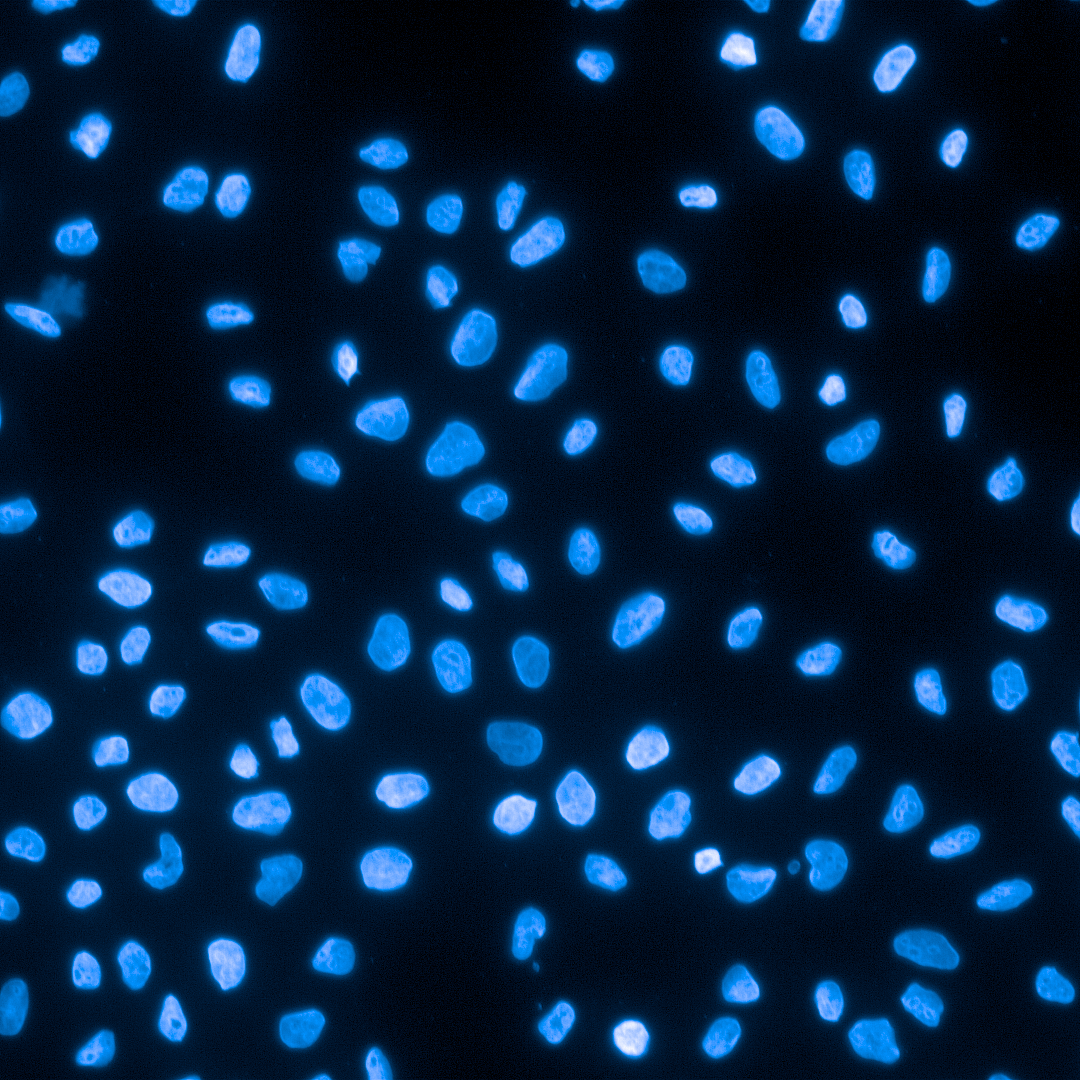

Supplement: Supplementary file 11 — Figure EV4 Source Data [file 44319_2025_514_MOESM11_ESM.zip › EV4/EV4C/DMSO_45_Hoechst.tif]

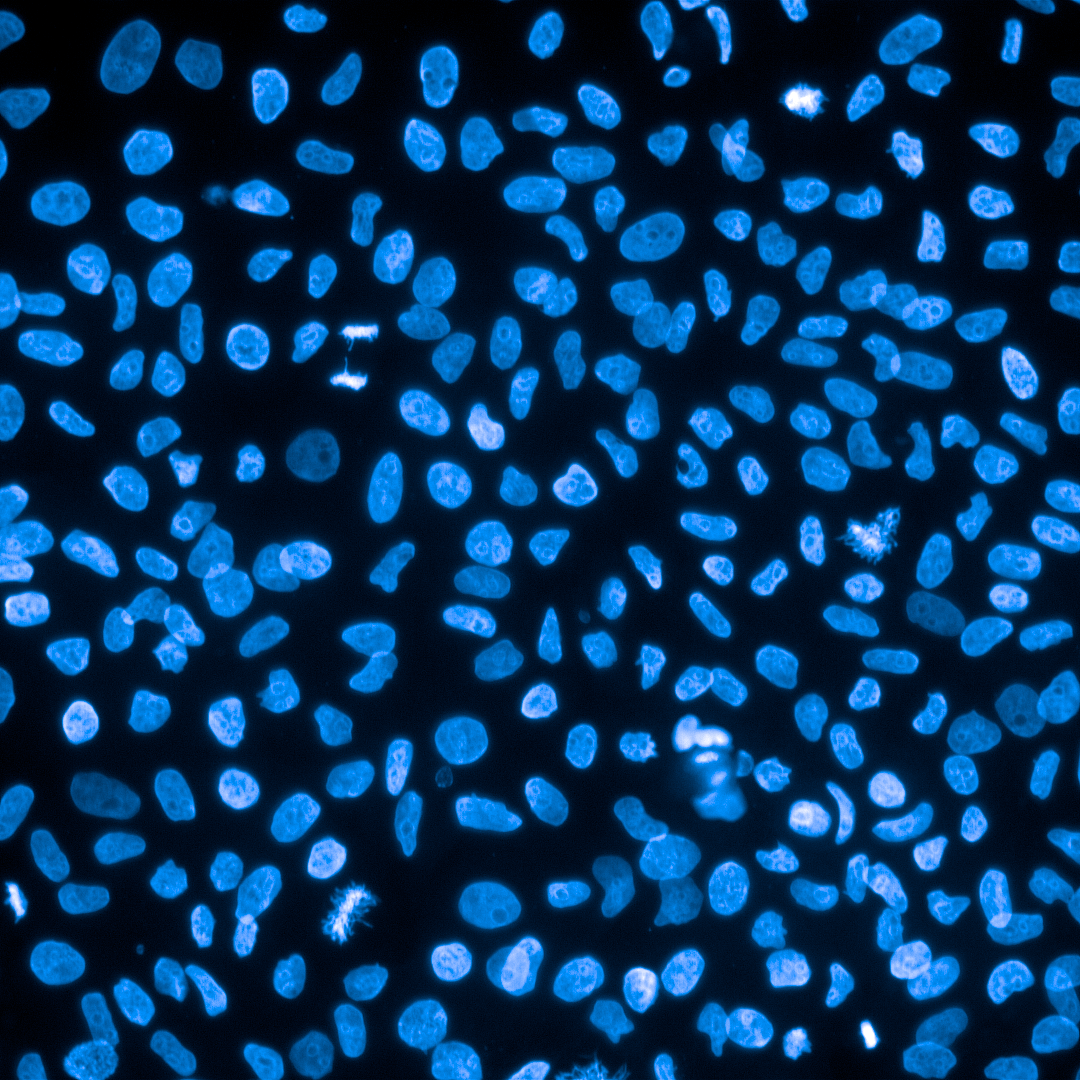

Supplement: Supplementary file 11 — Figure EV4 Source Data [file 44319_2025_514_MOESM11_ESM.zip › EV4/EV4C/YAP5SA_CHK1i_52_Hoechst.tif]

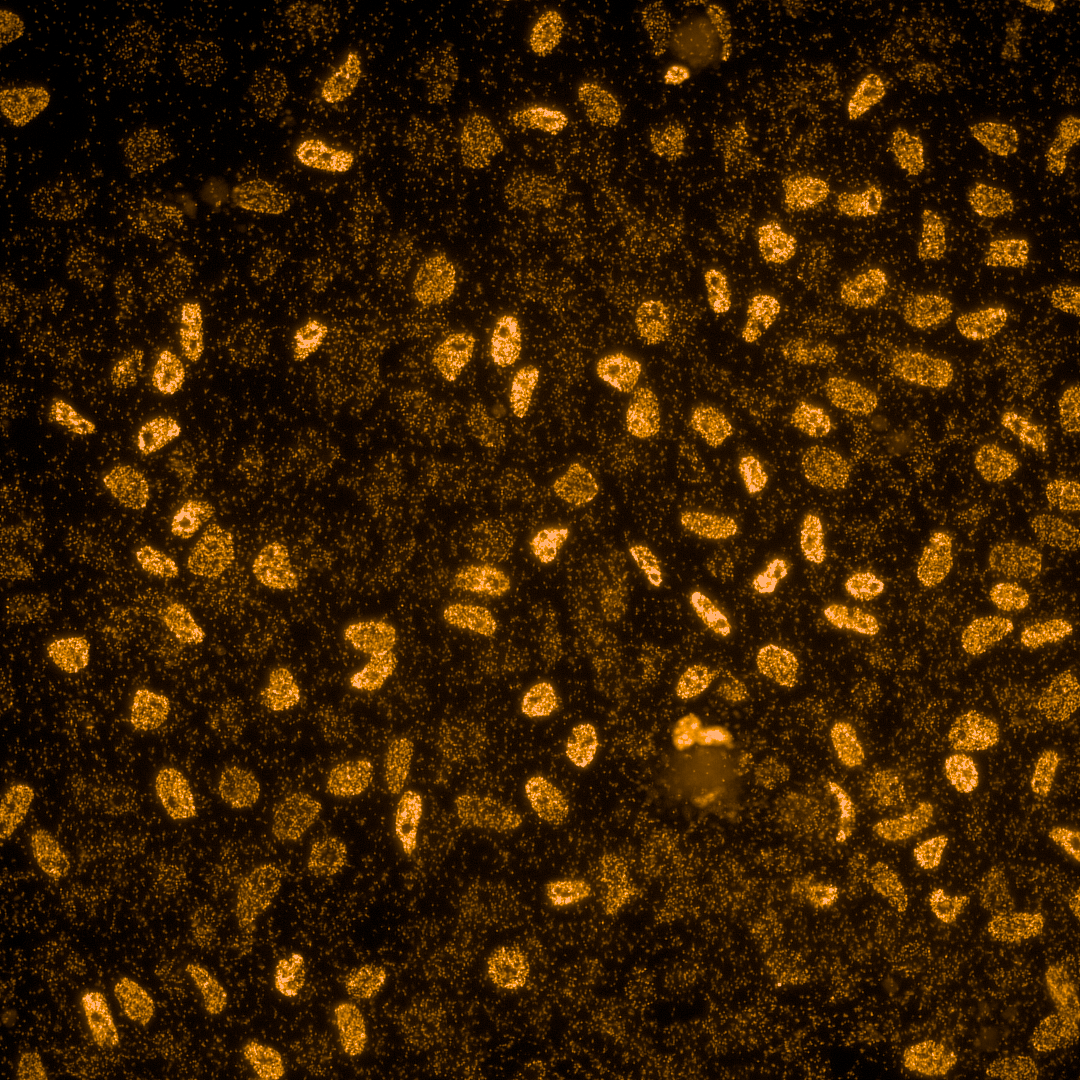

Supplement: Supplementary file 11 — Figure EV4 Source Data [file 44319_2025_514_MOESM11_ESM.zip › EV4/EV4C/YAP5SA_CHK1i_52_PLA.tif]

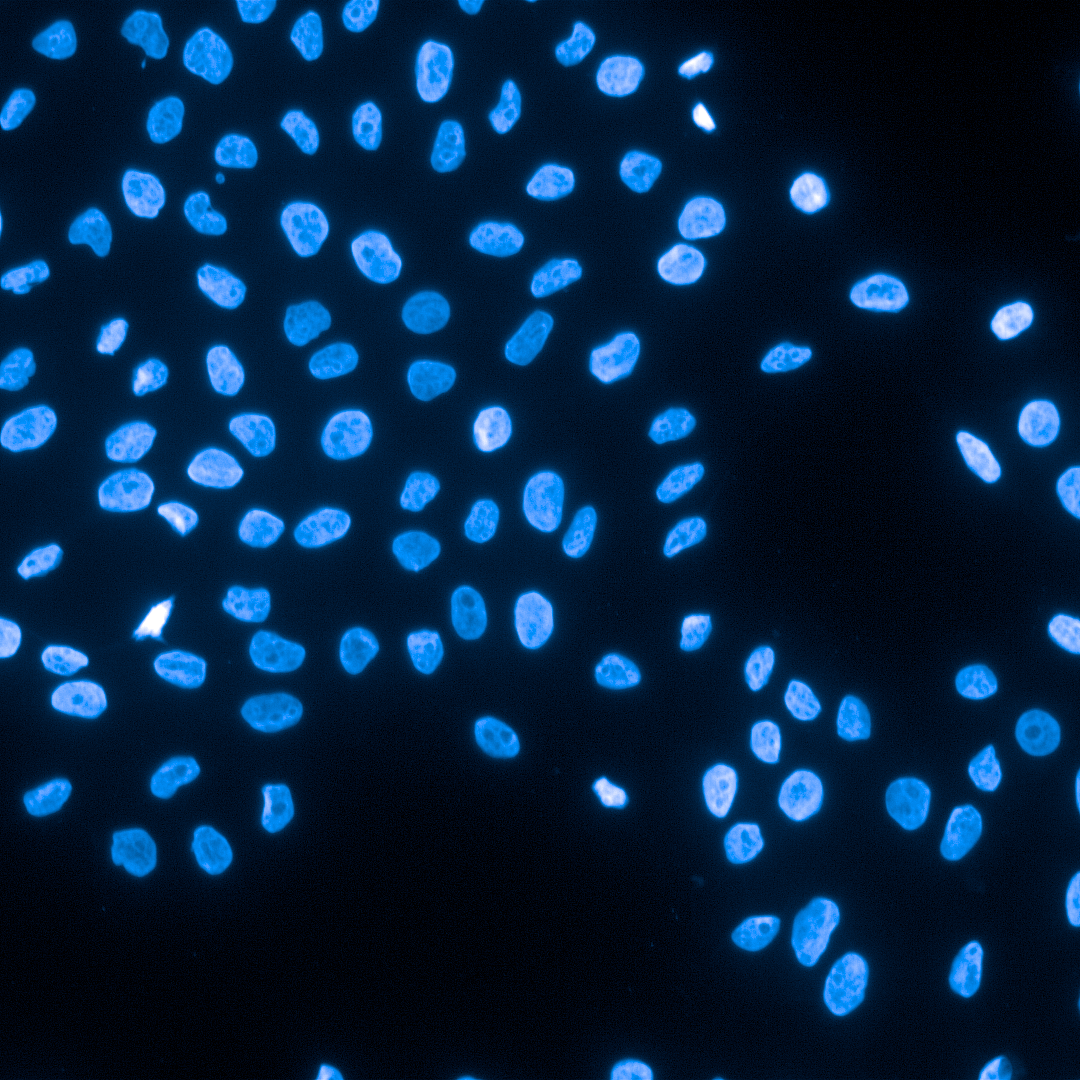

Supplement: Supplementary file 11 — Figure EV4 Source Data [file 44319_2025_514_MOESM11_ESM.zip › EV4/EV4C/RNAPolII_33_Hoechst.tif]

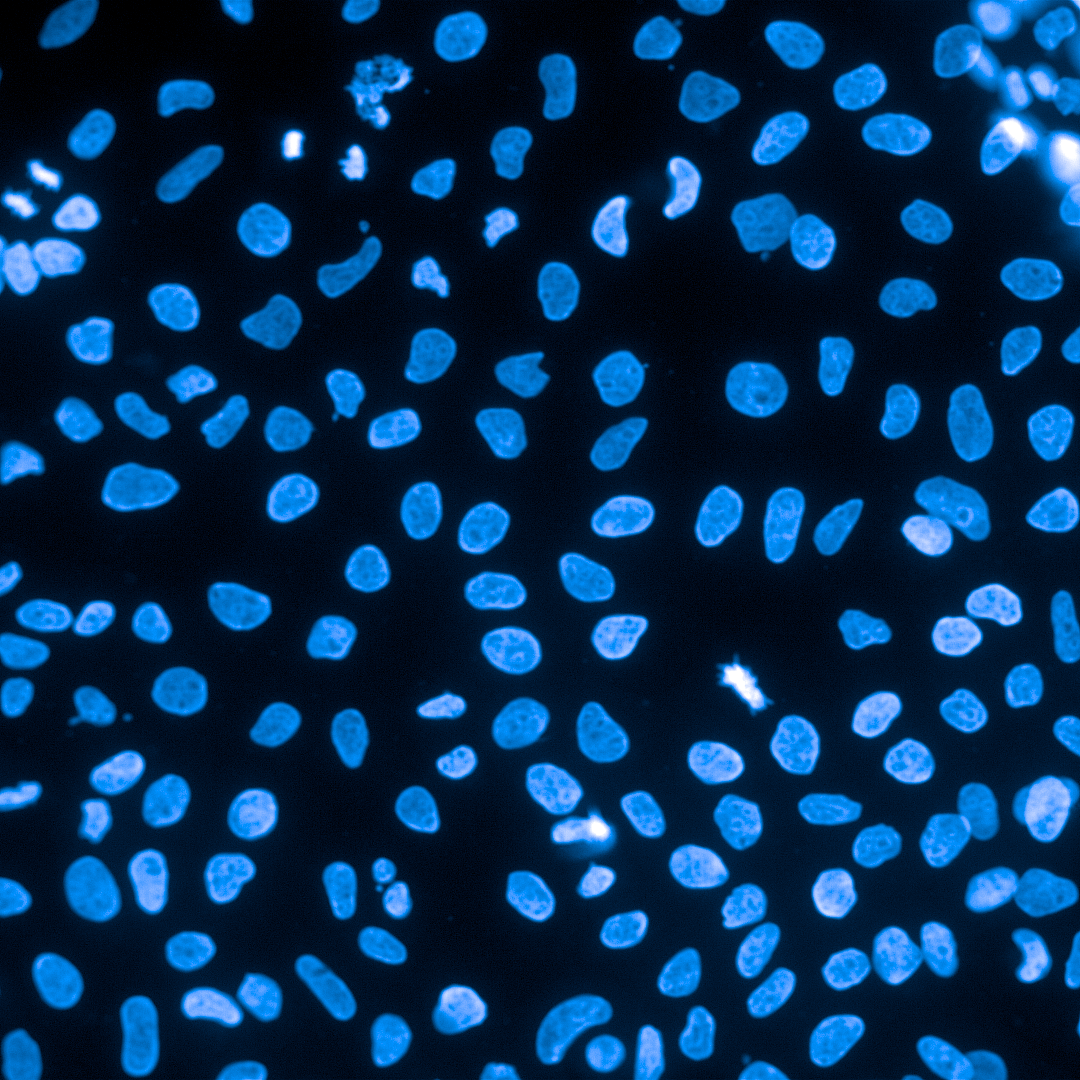

Supplement: Supplementary file 11 — Figure EV4 Source Data [file 44319_2025_514_MOESM11_ESM.zip › EV4/EV4C/YAP5SA_41_Hoechst.tif]

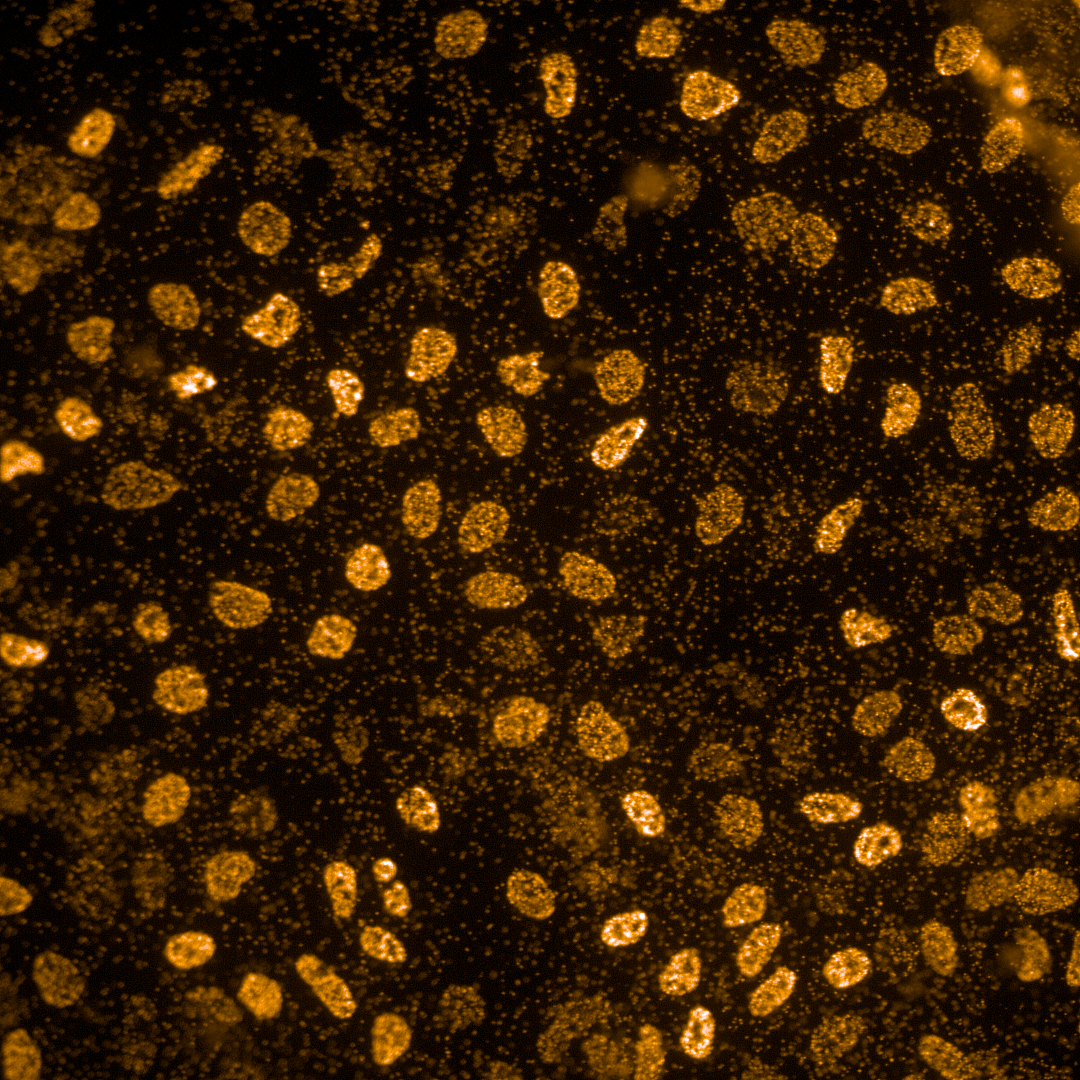

Supplement: Supplementary file 11 — Figure EV4 Source Data [file 44319_2025_514_MOESM11_ESM.zip › EV4/EV4C/YAP5SA_41_PLA.tif]

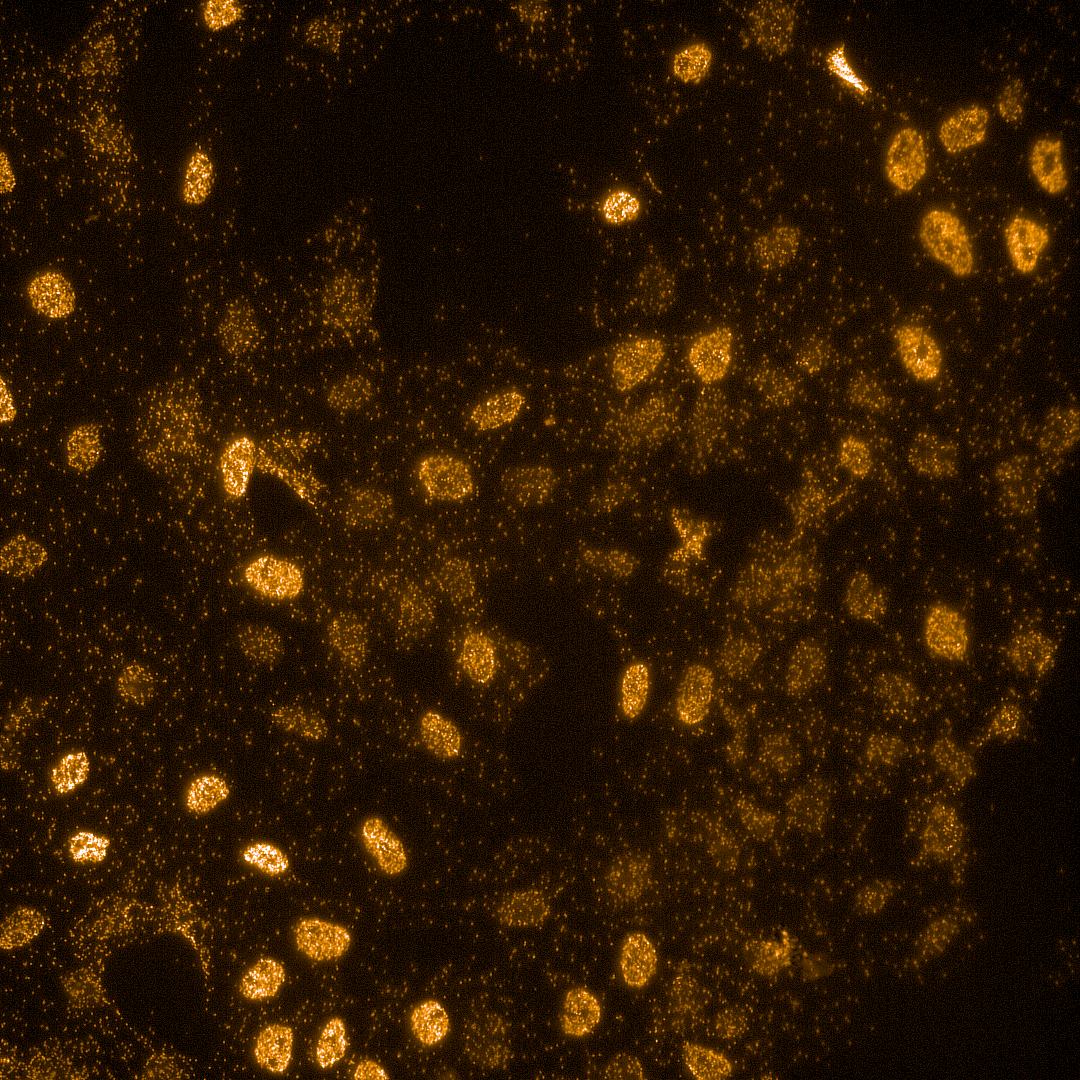

Supplement: Supplementary file 11 — Figure EV4 Source Data [file 44319_2025_514_MOESM11_ESM.zip › EV4/EV4C/CHK1i_40_PLA.tif]

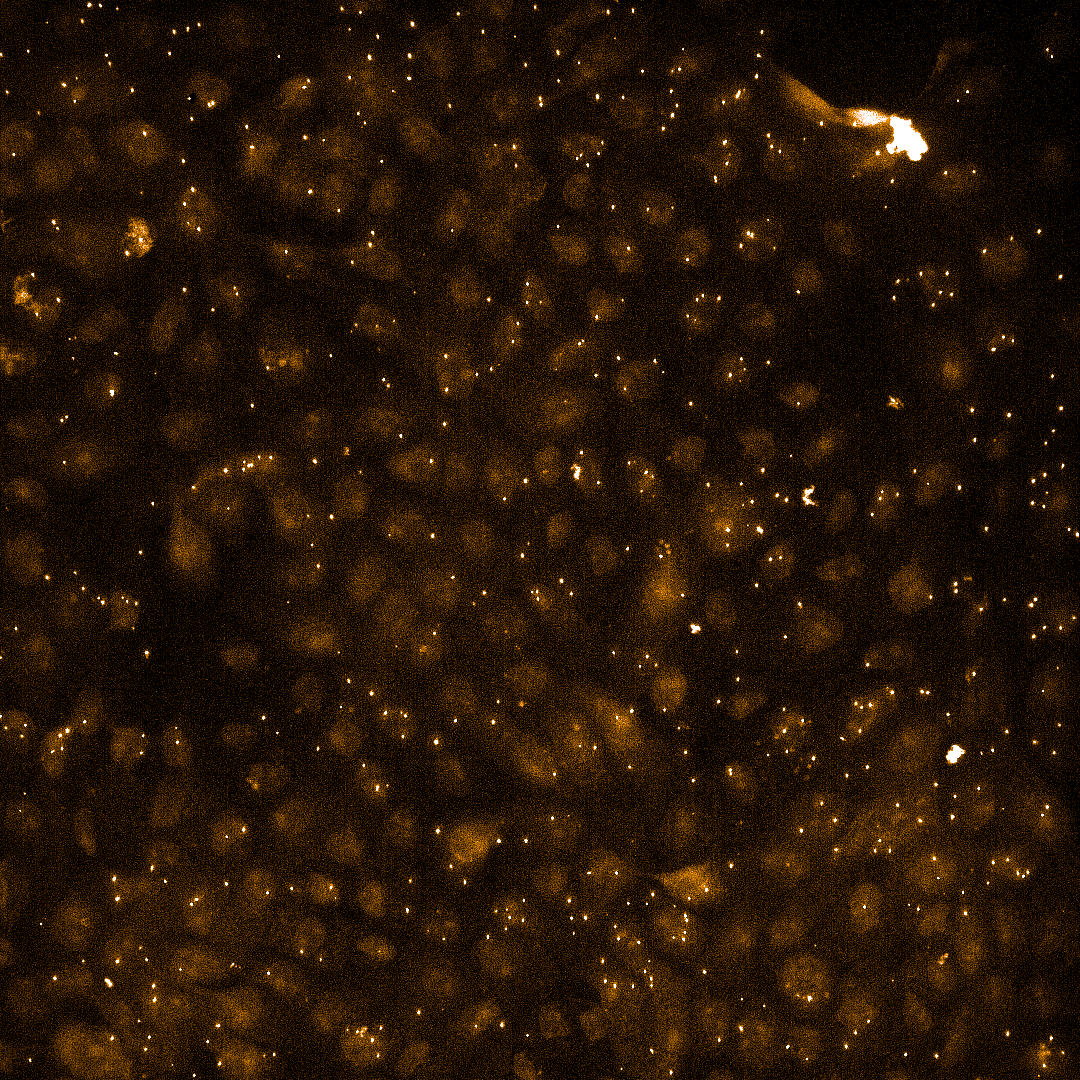

Supplement: Supplementary file 11 — Figure EV4 Source Data [file 44319_2025_514_MOESM11_ESM.zip › EV4/EV4C/PCNA_29_PLA.tif]

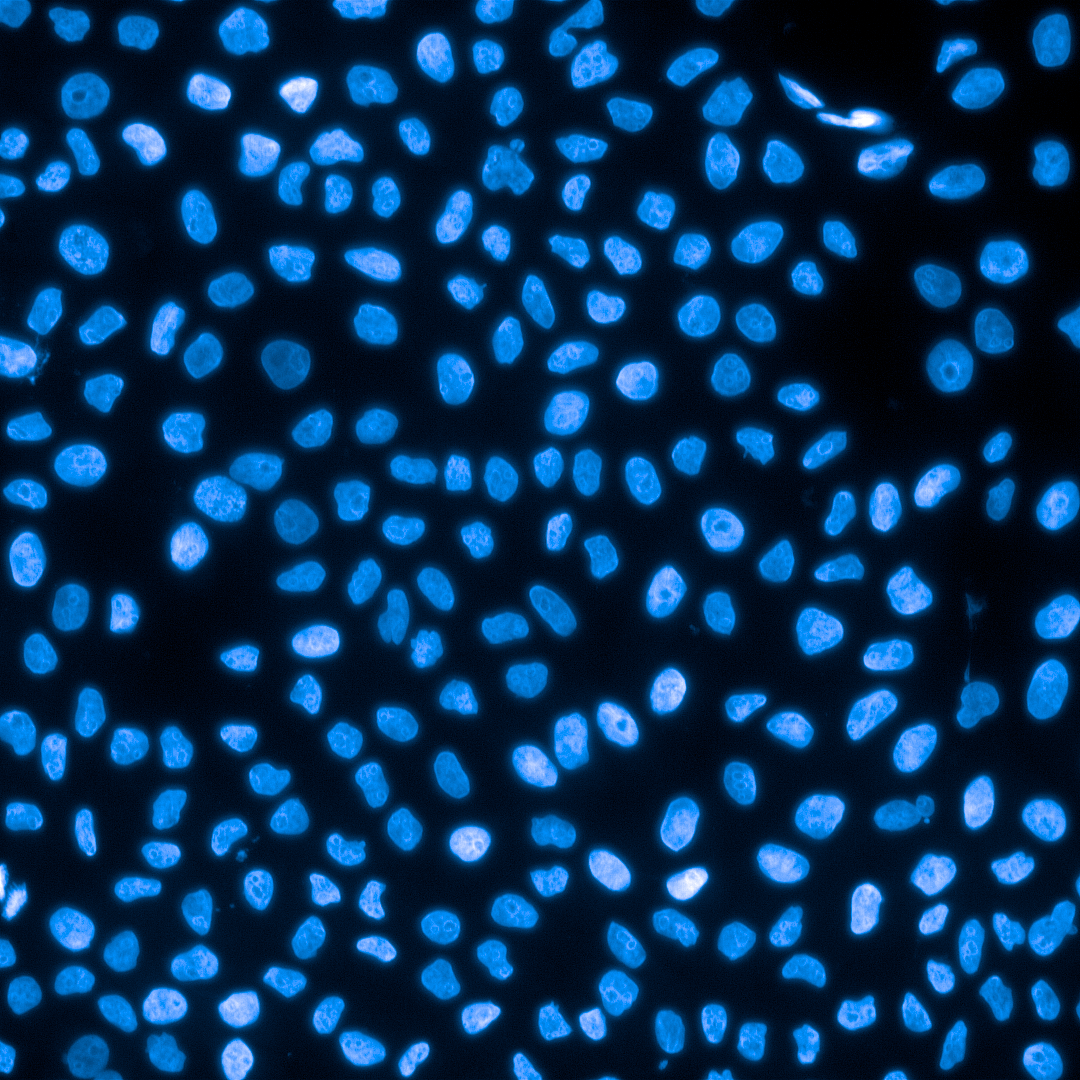

Supplement: Supplementary file 11 — Figure EV4 Source Data [file 44319_2025_514_MOESM11_ESM.zip › EV4/EV4C/PCNA_29_Hoechst.tif]

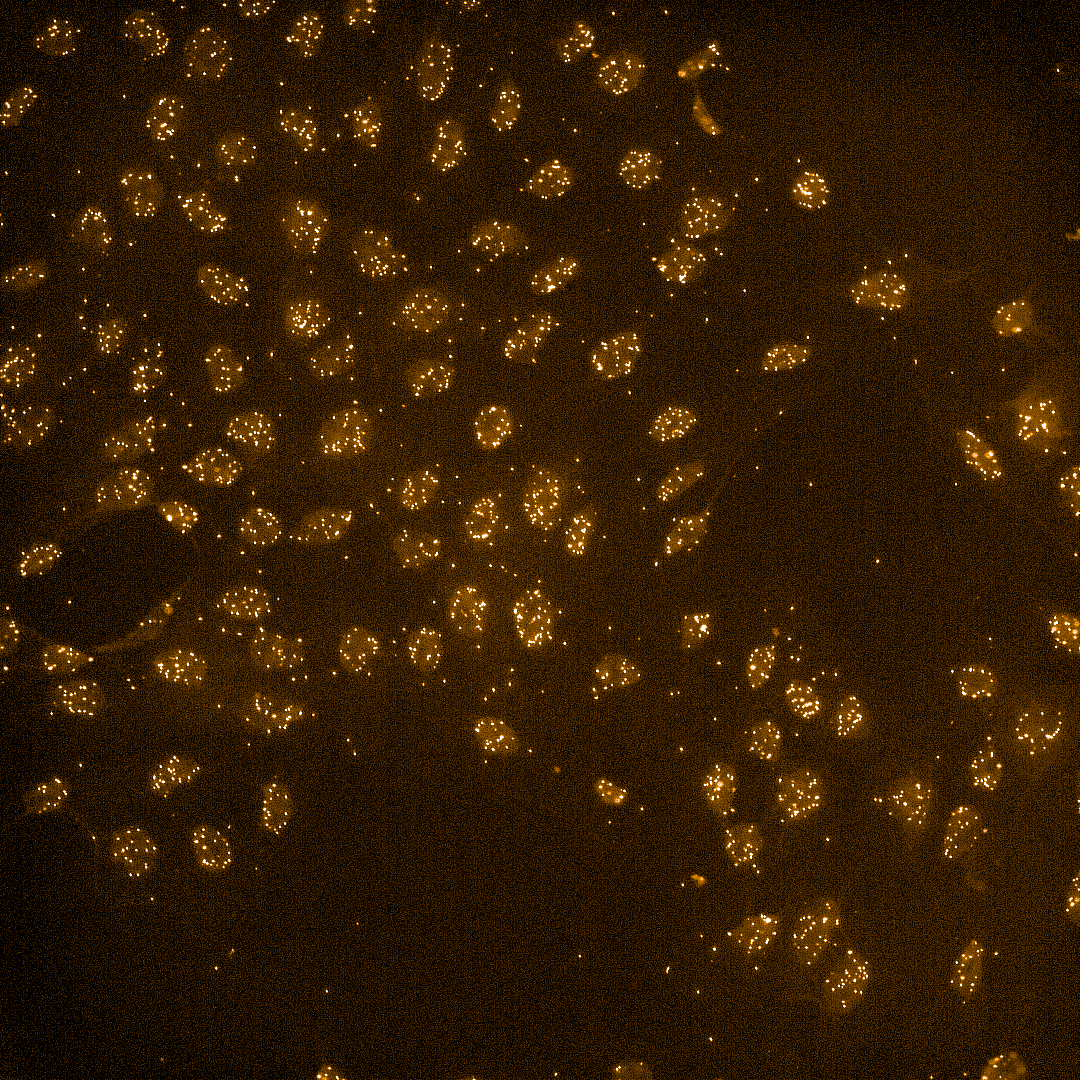

Supplement: Supplementary file 11 — Figure EV4 Source Data [file 44319_2025_514_MOESM11_ESM.zip › EV4/EV4C/RNAPolII_33_PLA.tif]

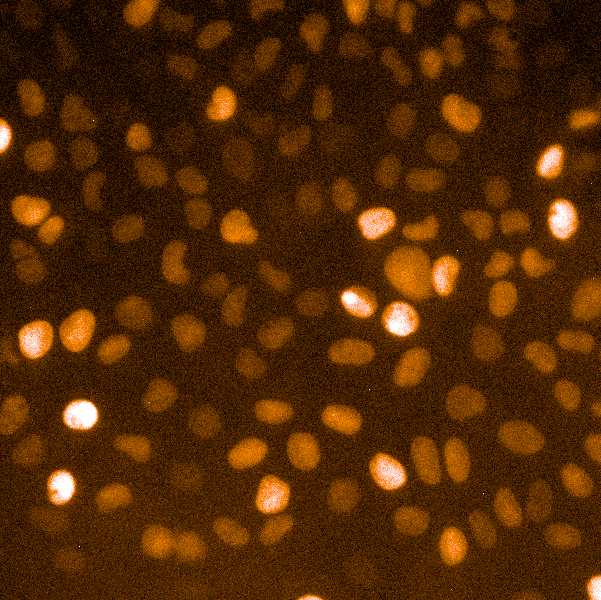

Supplement: Supplementary file 11 — Figure EV4 Source Data [file 44319_2025_514_MOESM11_ESM.zip › EV4/EV4B/mCherry.tif]

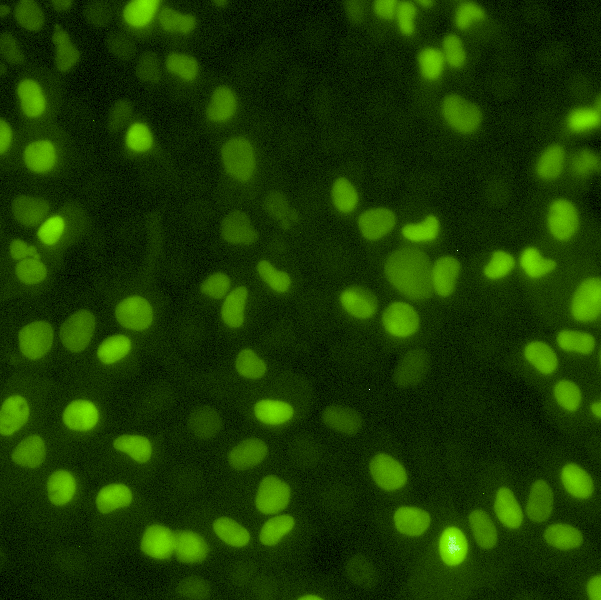

Supplement: Supplementary file 11 — Figure EV4 Source Data [file 44319_2025_514_MOESM11_ESM.zip › EV4/EV4B/mVenus.tif]

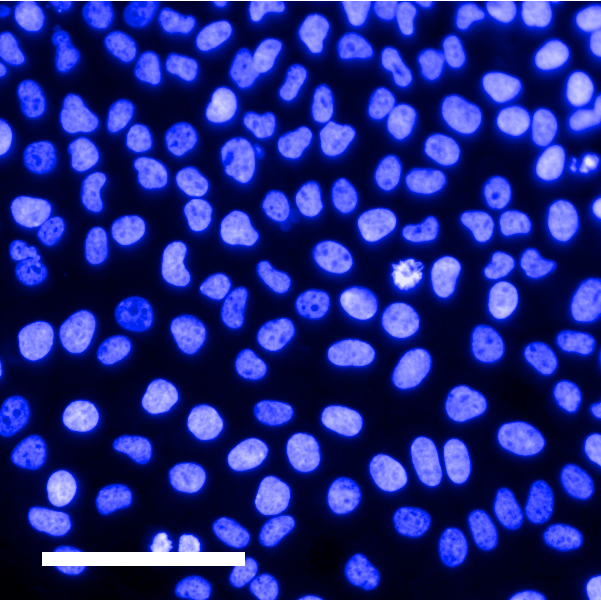

Supplement: Supplementary file 11 — Figure EV4 Source Data [file 44319_2025_514_MOESM11_ESM.zip › EV4/EV4B/Hoechst.tif]
