# Supplementary material for: Oncogenic YAP sensitizes cells to CHK1 inhibition via CDK4/6 driven G1 acceleration
Source: EMBO Rep. 2025 Jul 4;26(16):4017–39. doi: 10.1038/s44319-025-00514-5 (PMC12373906; doi:10.1038/s44319-025-00514-5)
Supplement: Supplementary file 12 — Expanded View Figures [file 44319_2025_514_MOESM12_ESM.pdf]

Expanded View Figures

Figure EV1. Oncogenic YAP activates CDK4/CDK6 in G1, but does not lead to CDK2 activation.

(**A**) Representative immunofluorescence images showing the activity-sensors for CDK2 (DHB-mVenus) and CDK4 (mCherry-CDK4KTR). Scale bar: 100 µm. (**B**) CDK4/6 and CDK2 activity during S and G2 phase in control and YAP5SA-expressing MCF10A cells. Violin plots display single-cell measurements from at least 2064 cells per condition. *P* values were calculated using unpaired Student's *t* test ($n = 3$ independent replicates). (**C**) CDK2 activity of control and YAP5SA-expressing MCF10A cells treated with increasing concentrations of palbociclib, measured by high-content microscopy using the CDK2 reporter construct. Data represent single-cell analysis from at least 1038 cells per condition from a representative experiment ($n = 3$ independent replicates). (**D**) Distribution of the pRB/RB ratio in S-phase. Violin plots display single-cell measurements from a representative experiment, with at least 1441 cells analyzed per condition. *P* values were calculated using ordinary one-way ANOVA ($n = 3$ independent replicates). (**E**) Density plots of the pRB/RB ratio for the conditions indicated in the legend. Kernel density estimation (KDE) was used to visualize the distribution of the pRB/RB ratios without binning. The density is normalized such that the area under each curve equals 1. (**F**) RT-qPCR analysis of the indicated G1 cyclins and CDKs. Data are shown as mean ± SD. Statistical significance was assessed using Student's *t* test ($n = 4$ independent replicates)). (**G**) Western blot analysis of the indicated G1 cyclins and CDKs. Actin served as a loading control. Source data are available online for this figure.

▶

© The Author(s)

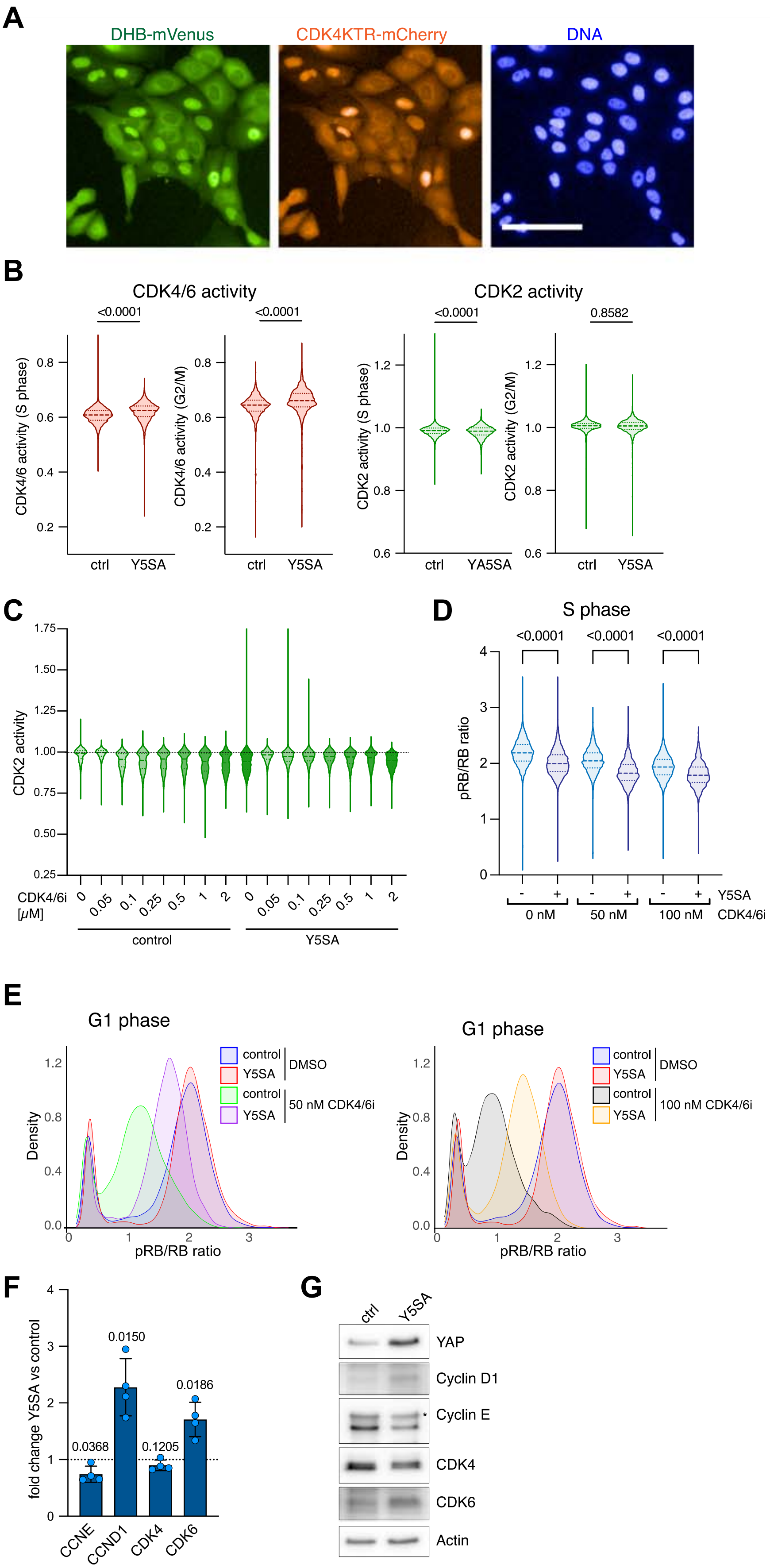
A
DHB-mVenus
CDK4KTR-mCherry
DNA
B
CDK4/6 activity
CDK2 activity
<0.0001
<0.0001
<0.0001
0.8582
CDK4/6 activity (S phase)
CDK4/6 activity (G2/M)
CDK2 activity (S phase)
CDK2 activity (G2/M)
ctrl Y5SA
ctrl Y5SA
ctrl YA5SA
ctrl Y5SA
C
CDK2 activity
CDK4/6i [μM]
0 0.05 0.1 0.25 0.5 1 2 0 0.05 0.1 0.25 0.5 1 2
control
Y5SA
D
S phase
<0.0001 <0.0001 <0.0001
pRB/RB ratio
- + - + - + Y5SA
0 nM 50 nM 100 nM CDK4/6i
E
G1 phase
G1 phase
control
Y5SA
DMSO
control
Y5SA
50 nM CDK4/6i
100 nM CDK4/6i
Density
pRB/RB ratio
F
fold change Y5SA vs control
0.0368
0.0150
0.1205
0.0186
CCNE CCND1 CDK4 CDK6
G
ctrl Y5SA
YAP
Cyclin D1
Cyclin E
CDK4
CDK6
Actin

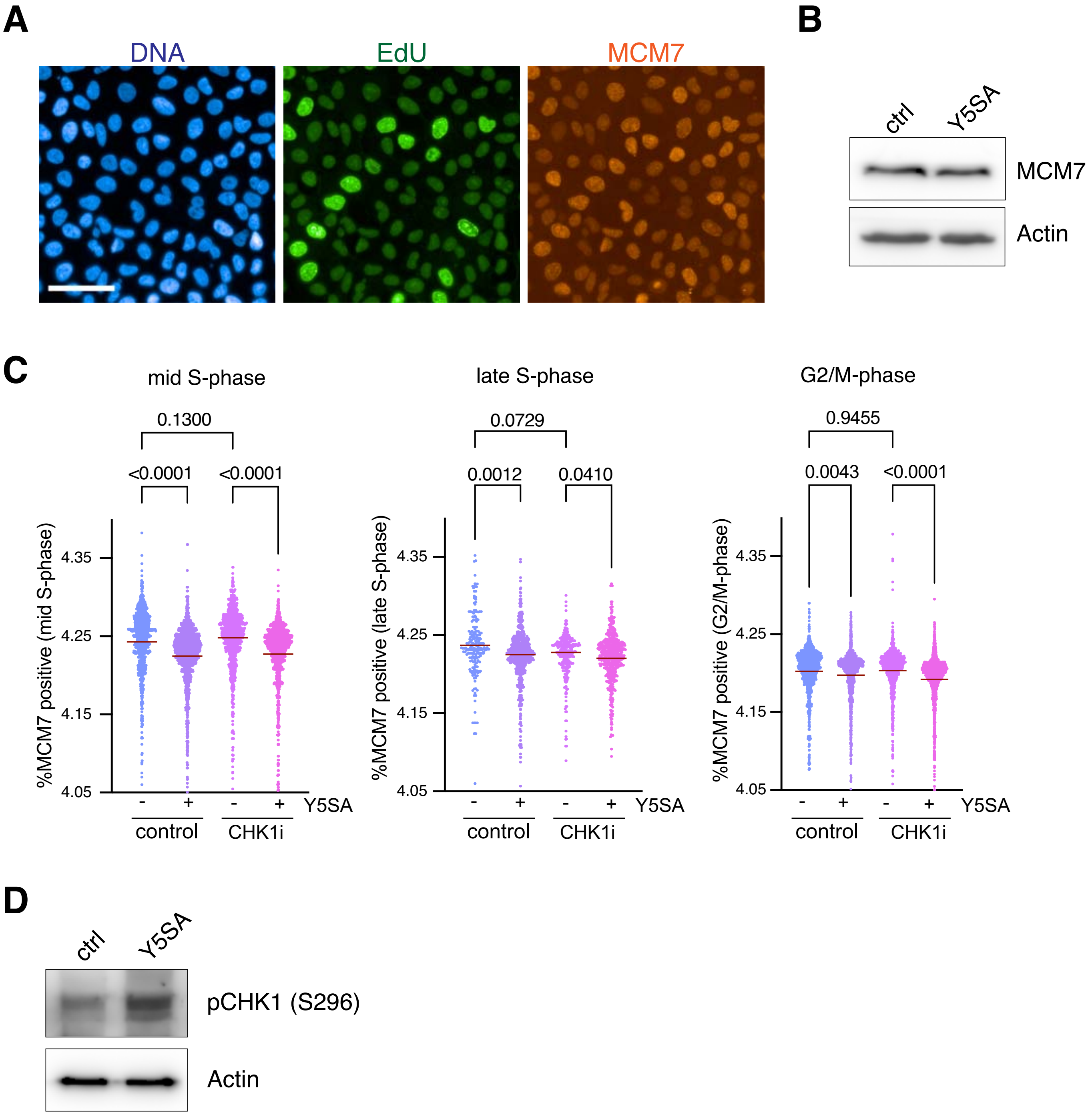

Figure EV2. YAP5SA does not alter MCM7 protein levels.

(**A**) Representative immunofluorescence image of MCM7 staining. Scale bar: 50 µm. (**B**) Immunoblot analysis to determine MCM7 levels. Actin served as a loading control. (**C**) Chromatin-bound MCM7 in mid and late S-phase and in G2. Violin plots display single-cell measurements from a representative experiment, with ≥204 cells analyzed per condition. *P* values were calculated using ordinary one-way ANOVA ($n = 3$ independent replicates). (**D**) Immunoblot analysis to determine pCHK1 (S296). Actin served as loading control. Source data are available online for this figure.

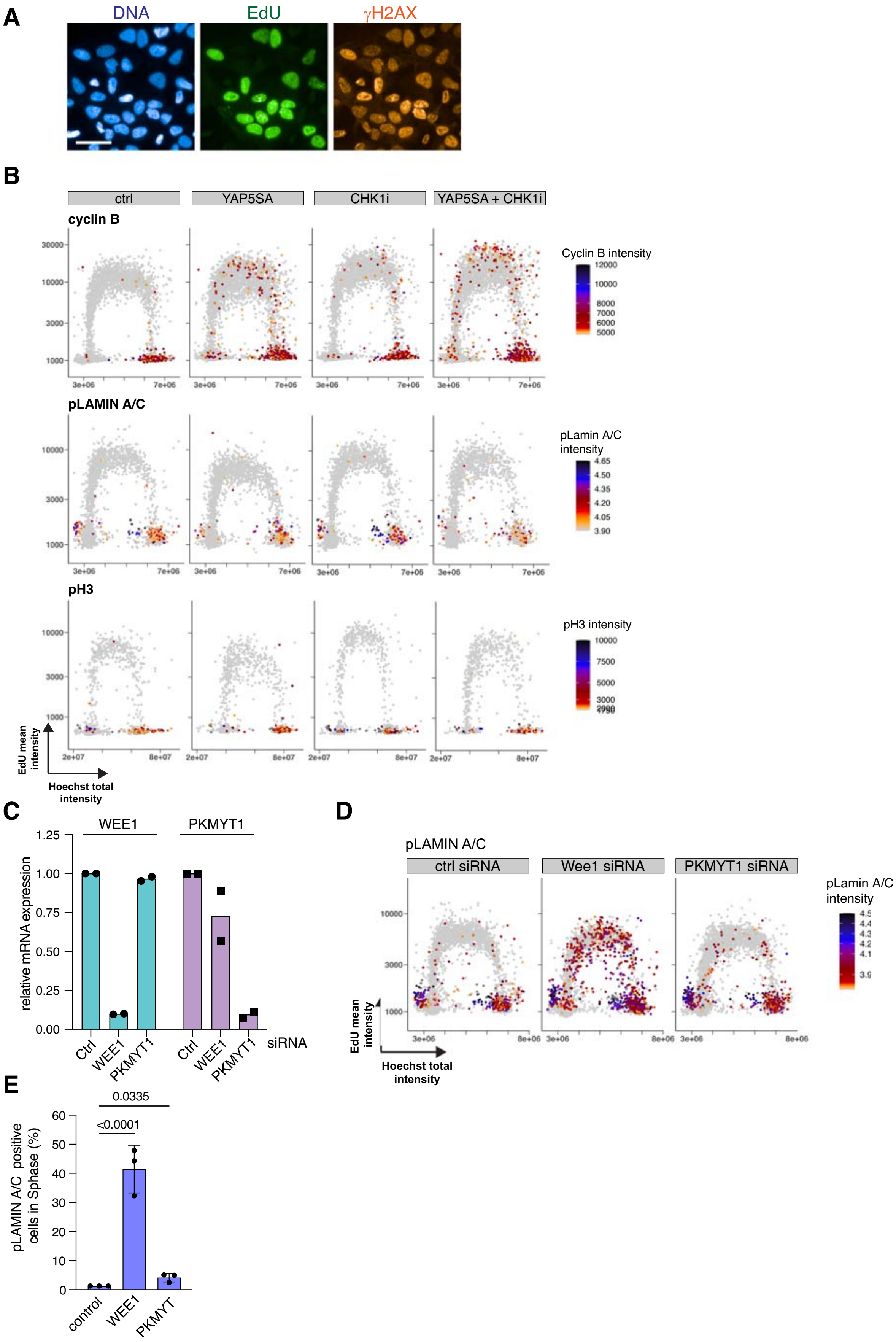
A
DNA
EdU
γH2AX
B
ctrl
YAP5SA
CHK1i
YAP5SA + CHK1i
cyclin B
Cyclin B intensity
pLAMIN A/C
pLamin A/C intensity
pH3
pH3 intensity
EdU mean intensity
Hoechst total intensity
C
WEE1
PKMYT1
relative mRNA expression
Ctrl
WEE1
PKMYT1
siRNA
D
pLAMIN A/C
ctrl siRNA
Wee1 siRNA
PKMYT1 siRNA
pLamin A/C intensity
E
0.0335
<0.0001
pLAMIN A/C positive cells in Sphase (%)
control
WEE1
PKMYT

© The Author(s)

Figure EV3. CHK1 inhibition in YAP5SA-expressing cells does not lead to premature mitosis in S-phase.

(**A**) Representative immunofluorescence images of γH2AX immunostaining. Scale bar: 50 μm. See also Fig. 4B. (**B**) High-content microscopy-based analysis of cytoplasmic cyclin B, pLAMIN A/C(S22) and pH3(S10). S-phase cells were labeled with EdU. For each panel, the following number of cells were randomly selected: cyclin B: 5000 cells, pLamin: 3500 cells, pH3: 1600 cells ($n = 3$ independent replicates). (**C**) RT-qPCR was used to validate the siRNA-mediated knockdown of WEE1 and PKMYT1 ($n = 2$ independent replicates). (**D**) High-content microscopy-based analysis of pLAMIN A/C(S22) in cells transfected with WEE1 and PKYT1 specific siRNAs or with a control siRNA. For each sample, 4000 cells were randomly selected ($n = 3$). (**E**) Quantification of the percentage of cells positive for pLAMIN A/C(S22) in S-Phase in the experiment shown in (**D**). Mean +/− SD. *P* values were calculated using ordinary one-way ANOVA ($n = 3$ independent replicates). Source data are available online for this figure.

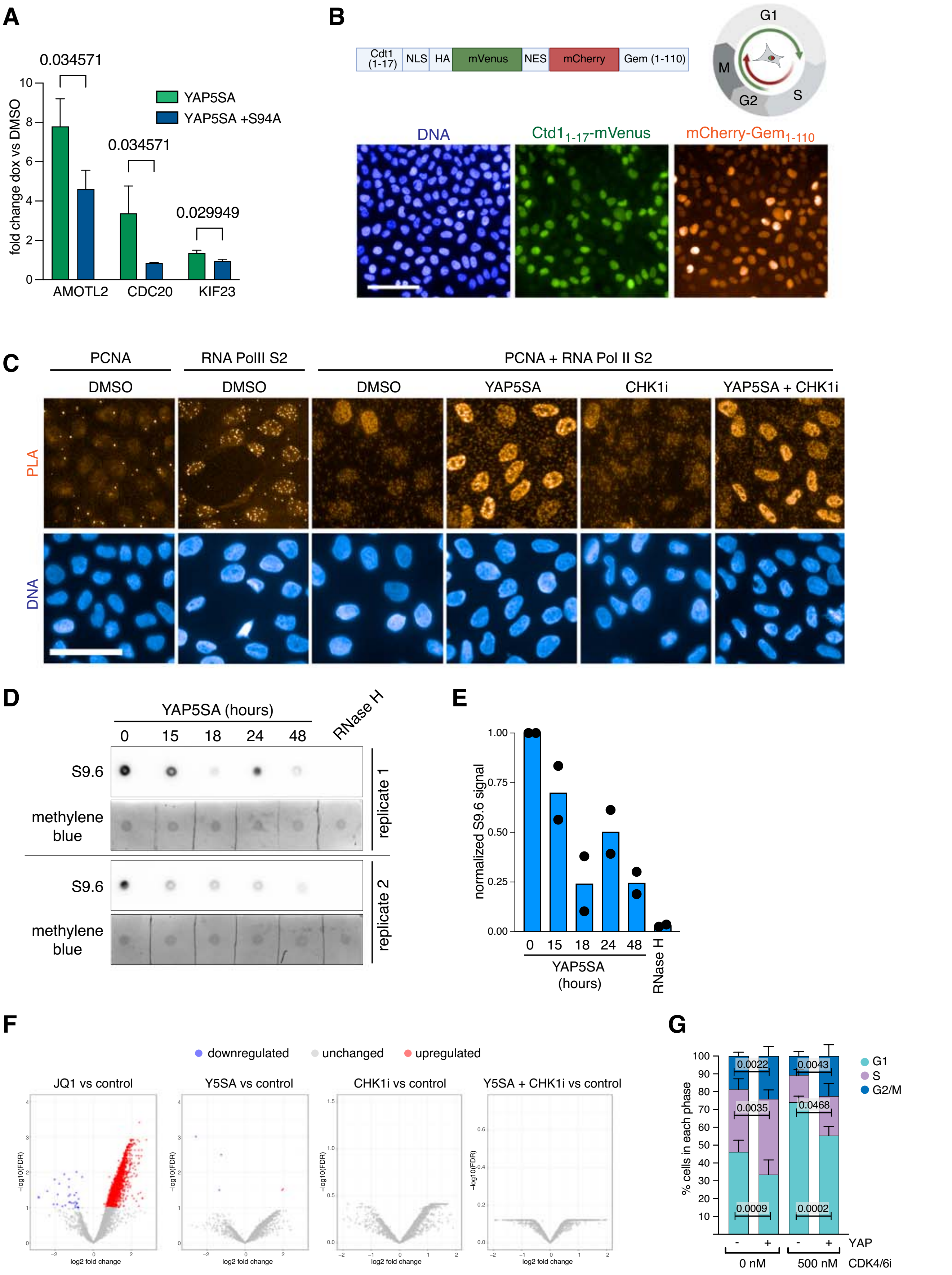
A
0.034571
0.034571
0.029949
YAP5SA
YAP5SA +S94A
fold change dox vs DMSO
AMOTL2
CDC20
KIF23
B
Cdt1 (1-17)
NLS
HA
mVenus
NES
mCherry
Gem (1-110)
G1
M
G2
S
DNA
Ctd1 1-17-mVenus
mCherry-Gem 1-110
C
PCNA
RNA PolII S2
PCNA + RNA Pol II S2
DMSO
DMSO
DMSO
YAP5SA
CHK1i
YAP5SA + CHK1i
PLA
DNA
D
YAP5SA (hours)
0
15
18
24
48
RNase H
S9.6
methylene blue
replicate 1
S9.6
methylene blue
replicate 2
E
normalized S9.6 signal
YAP5SA (hours)
RNase H
F
downregulated
unchanged
upregulated
JQ1 vs control
Y5SA vs control
CHK1i vs control
Y5SA + CHK1i vs control
-log10(FDR)
log2 fold change
G
0.0022
0.0043
0.0035
0.0468
0.0009
0.0002
% cells in each phase
G1
S
G2/M
YAP
0 nM
500 nM
CDK4/6i

 © The Author(s)

Figure EV4. YAP5SA enhances the proximity between the transcription and replication machinery but does not lead to increased R-loop levels.

(**A**) RT-qPCR was used to analyze expression of the indicated genes upon expression of YAP5SA or YAP5SA-S94A. Mean +/− SD. *P* values were calculated using an unpaired Student's *t* test ($n = 3$ independent replicates). (**B**) Scheme of the PIP-FUCCI cell cycle sensors and representative images of MCF10A-YAP5SA cells expressing the sensors. Scale bar: 100 µm. (**C**) Representative images of Proximity Ligation assay (PLA) between PCNA and RNA Pol II phosphorylated at Serine 2. Scale bar: 50 µm. See Fig. 5I. (**D**) Dot blot assay to detect R-loops in control MCF10A cells and cells expressing YAP5SA for the indicated times. Total DNA was analyzed with the S9.6 monoclonal antibody, which recognizes DNA:RNA hybrids. RNase H1 treatment served as a control. Methylene blue staining was used as a control for equal loading ($n = 2$ independent replicates). (**E**) Quantification of the S9.6 dot blot signal in (**D**) normalized to total DNA detected by methylene blue staining ($n = 2$ independent replicates). (**F**) MapR was used to assess R-loop levels in control MCF10A cells and YAP5SA-expressing cells control treated or after treatment with CHK1i. Treatment with the BRD4 inhibitor JQ1, which has been shown to increase R-loop levels, served as positive control. MapR peaks were called by MACS2. The volcano plot shows differential R-loops as identified by DiffBind ($n = 2$ independent replicates). (**G**) Fraction of cells in each phase of the cell cycle with and without treatment with 500 nM palbociclib for 24 h. Mean +/− SD. *P* values were calculated using ordinary one-way ANOVA ($n = 5$ independent replicates). Source data are available online for this figure.
